# Positive Catch & Economic Benefits of Periodic Octopus Fishery Closures: Do Effective, Narrowly Targeted Actions ‘Catalyze’ Broader Management?

**DOI:** 10.1371/journal.pone.0129075

**Published:** 2015-06-17

**Authors:** Thomas A. Oliver, Kirsten L. L. Oleson, Hajanaina Ratsimbazafy, Daniel Raberinary, Sophie Benbow, Alasdair Harris

**Affiliations:** 1 Blue Ventures Conservation, 39–41 North Road, London, N7 9DP, United Kingdom; 2 Department of Biology, University of Hawaiʻi Mānoa, 2853 McCarthy Mall, Edmondson Hall 309, Honolulu, Hawaii, 96822, United States of America; 3 Department of Natural Resources and Environmental Management, University of Hawaiʻi Mānoa, 1910 East West Road, Sherman 101, Honolulu, Hawaii, 96822, United States of America; 4 Environmental Change Institute, University of Oxford, South Parks Road, Oxford, OX1 3QY, United Kingdom; University of Windsor, CANADA

## Abstract

**Overview:**

Eight years of octopus fishery records from southwest Madagascar reveal significant positive impacts from 36 periodic closures on: (a) fishery catches and (b) village fishery income, such that (c) economic benefits from increased landings outweigh costs of foregone catch. Closures covered ~20% of a village’s fished area and lasted 2-7 months.

**Fishery Catches from Each Closed Site:**

Octopus landings and catch per unit effort (CPUE) significantly increased in the 30 days following a closure’s reopening, relative to the 30 days before a closure (landings: +718%, p<0.0001; CPUE: +87%, p<0.0001; n = 36). Open-access control sites showed no before/after change when they occurred independently of other management (“no ban”, n = 17/36). On the other hand, open-access control sites showed modest catch increases when they extended a 6-week seasonal fishery shutdown (“ban”, n = 19/36). The seasonal fishery shutdown affects the entire region, so confound all potential control sites.

**Fishery Income in Implementing Villages:**

In villages implementing a closure, octopus fishery income doubled in the 30 days after a closure, relative to 30 days before (+132%, p<0.001, n = 28). Control villages not implementing a closure showed no increase in income after “no ban” closures and modest increases after “ban” closures. Villages did not show a significant decline in income during closure events.

**Net Economic Benefits from Each Closed Site:**

Landings in closure sites generated more revenue than simulated landings assuming continued open-access fishing at that site (27/36 show positive net earnings; mean +$305/closure; mean +57.7% monthly). Benefits accrued faster than local fishers’ time preferences during 17-27 of the 36 closures. High reported rates of illegal fishing during closures correlated with poor economic performance.

**Broader Co-Management:**

We discuss the implications of our findings for broader co-management arrangements, particularly for catalyzing more comprehensive management.

## Introduction

As over-exploitation and global change threaten reefs worldwide, sustainably managing coral reefs is crucial to protecting both reef biodiversity and the food security of hundreds of millions of coastal people [1–4]. Because two-thirds of all reefs lie in developing countries [5], the goal of conserving reefs globally requires management strategies that can effectively balance both conservation and development goals. This developing world setting frequently includes high population growth rates, low incomes, and weak national-scale governance [6–8]. In this context, local communities’ support for management actions is crucial to effectively protect biodiversity and human livelihoods [9–13].

There is a growing body of research reporting on coastal management efforts designed, enforced, and maintained by communities or communities with an external partner (co-management) [9,14–16]. Employing a broad array of measures, community and co-management arrangements around the world have produced positive outcomes for both conservation and development goals [9,13,16]. When effective, such arrangements can help communities better manage their resources over the long term, helping them break from the tragedy of the commons, where open access leads to overexploitation, and from resource-dependent poverty traps, where natural resource depletion and dependence reinforce each other [17–19]. However, while community and co-management models are becoming more common, quantitative impact assessments remain uncommon and many management failures are under-reported. These research gaps hinder robust generalizations about the effectiveness of community and co-management approaches [9,11].

The *periodic fishery closure*, in which fishers temporarily refrain from harvesting in specific areas [20,21] is an increasingly popular community-based tool with a growing base of empirical support [22–24]. Periodic closures have long been a part of traditional fishing cultures across the Indo-Pacific [20,25–27], and still play an active role in community management of marine resources in the region [22–24,28]. Periodic harvest, or pulse fishing, also has been a commonly discussed strategy in the western fisheries literature [29], and has been suggested as a viable alternative to constant, or stationary, fishing yields since at least the 1970s [30,31].

Many periodic harvest regimes have been designed with a single-species in mind [32]. Practical examples from both models and field data generally target sedentary marine invertebrates, and highlight that urchins [33], sea scallops [34,35], and abalone [36] make good candidates for a periodic regime. Periodic closure regimes in the tropical Indo-Pacific have shown positive effects on abundance in giant clams (*Tridacna spp*.) [22] and varied results for trochus (aka topshell, *Tectus niloticus*) [22–24,37,38].

Periodic harvest strategies in artisanal contexts frequently apply not to single target species but instead to multi-species assemblages, including relatively long-lived reef fishes [22–24,28,39,40]. The few studies that have shown positive effects of periodic closures on mixed reef fish fisheries noted increases in fish biomass in periodically closed areas relative to open access sites [22,23]. All three focal areas in these studies, however, were characterized by small human populations exerting low fishing pressure on resources to which they have exclusive tenure [22,23]; the results do not hold in areas subject to higher fishing pressures [28,39–41], perhaps because closure periods were too short, open periods too long, or fishing intensity during open periods was too intense to support robust recovery from fishing mortality [28,39–41]. Another reason may be that in areas with higher pressure and competition, fisher populations prefer immediate reward from landing a smaller catch today over a larger and more uncertain future catch [42].

While results from the field have been variable, models of fisheries economics suggest that in certain cases a periodic harvest can provide a better economic yield than stationary harvest [30,31], specifically when the fishery has low selectivity [31,43]. A fishery’s optimal opening/closing cycle (i.e., the pulse-length) is a function of both the target species’ biology (i.e., specifically the target species’ growth rate and life-span) and the fishery’s economics (i.e., landing prices and the local fisher’s discount rate, their time preference for immediate versus delayed reward) [43]. The time between openings varies dramatically depending on the target species’ biology [32], and higher discount rates lead to either shortening the optimal closure durations or shifting the economic optimum to stationary, rather than periodic harvest [31,44].

Models and experience suggest that the success or failure of a periodic closure regime depends on the governance system’s ability to match fishing patterns to a fishery’s “optimal” periodic harvest schedule [23,24,29]. Factors shown to improve the odds of matching actual and optimal harvest in the context of periodic closures include: exclusive tenure to the resource in question, respected and legitimate leadership, high social capital, low fishing pressure, low efficiency gears, and robust ecological knowledge [22–24,45]. Not surprisingly, these governance factors mirror those that more generally correlate with successful community/co-management [13,16,46].

Experience with successful, targeted management might also serve as a catalyst for broader community management [24,45,47]. In Vanuatu, government-sponsored management efforts employing a range of interventions, including periodic closures, led to community engagement with managers and co-management of many other species of fish and invertebrates [47]. In Indonesia, villages with active or lapsed periodic closure traditions showed broader, more active marine management than villages with no such tradition [45]. Commons theory suggests that communities are more likely to engage in management when expected benefits outweigh the perceived costs of management [17]. In these cases, successful demonstrations of desirable benefit:cost ratios likely informed expectations, while offering an opportunity to build governance capacity and social capital needed to broaden management efforts [17].

**Fig 1:**
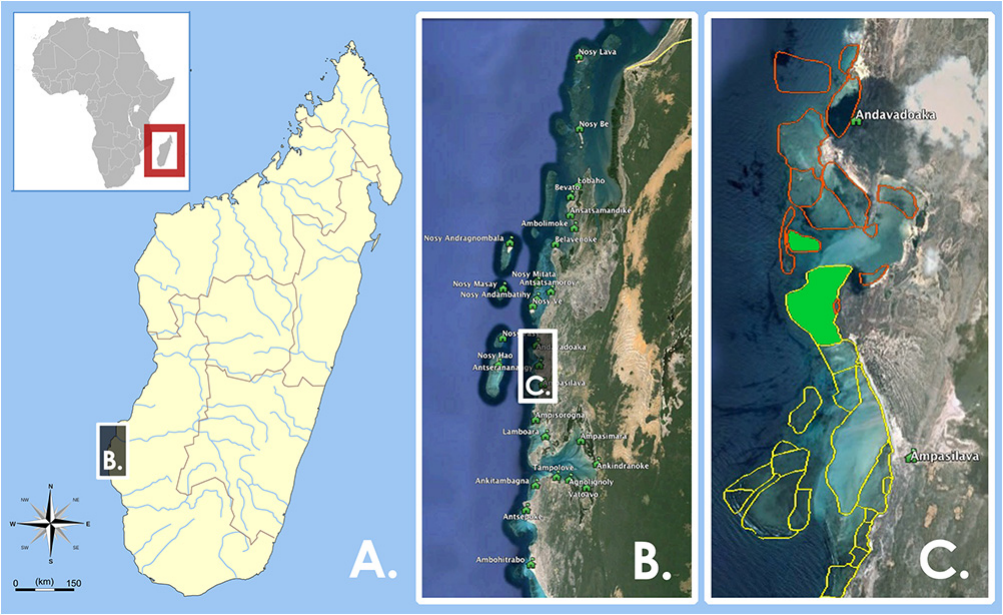
Maps of Study Area. (A) Large-scale map of Madagascar and the African continent, (B) Inset of the 25 villages of the Velondriake Locally Managed Marine Area in southwestern Madagascar. Vertical box extent is ~75 km. (C) Representative example of a periodic octopus fishery closure. Indicated in the map are two villages, Andavadoaka and Ampasilava, with their respective octopus fishing sites mapped in orange and yellow. In green, you can see the sites Amagnahitse and Nosinkara, in which these two villages have repeatedly co-implemented a periodic octopus fishery closure.

Here we present an analysis of the fishery and economic effects of periodic octopus fishery closures in the Velondriake Locally Managed Marine Area (LMMA) in southwest Madagascar (Fig 1). This work serves to fill research gaps by providing empirical impact assessments of co-management outcomes for a specific periodic fishery closure regime. Establishing the baseline efficacy of these interventions is particularly timely, as the use of periodic closures as a fisheries management tool is proliferating across the western Indian Ocean [48].

**Fig 2:**
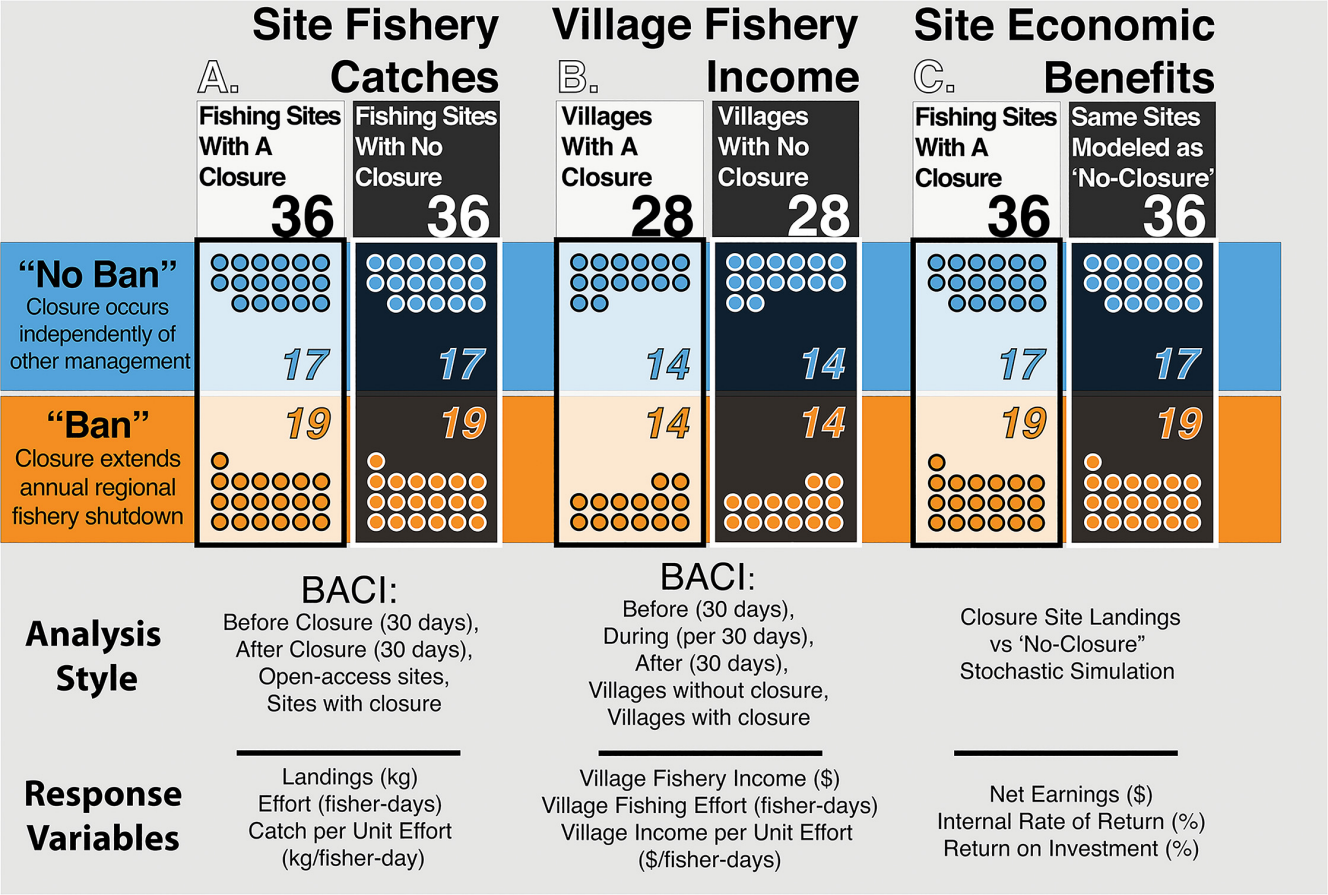
Experimental design and samples sizes used to investigate effects of periodic fishery closures. (A) Site fishery catches, (B) Village fishery income, and (C) Site net economic benefts. Colors highlight the distinctions among “no-ban” and “ban” closures, and between closure sites/villages and either open-access controls (A&B) or simulated landings (C).

To do so, first, we quantify effects on site-specific landings and catch per unit effort (CPUE) from multiple periodic closure events compared to paired controls. Second, we examine octopus fishery-generated income accrued at the village level. Third, we assess whether individual closed sites generate net economic benefits, and compare the rates at which these benefits are generated to local fishers’ time preferences (Fig 2). Finally, as broader co-management efforts in the LMMA followed the widespread adoption of the octopus closure regime, we discuss a fertile area for future research testing the hypothesis that effective periodic closures can serve as a catalyst for broader community management.

## Methods

### 2.1. Marine Resource Management in the Velondriake LMMA

Starting in 2003, the non-governmental organization Blue Ventures, with local and international partners (Institut Halieutique et des Sciènces Marines, Wildlife Conservation Society, COPEFRITO) began a series of meetings with the community of Andavadoaka in southwest Madagascar to discuss approaches to managing local marine resources. In initial conversations, the community demurred from engaging in permanent no-take areas, but was willing to attempt a 7-month closure of octopus fishing on a shallow offshore reef beginning November 1, 2004 [49].

After a favorable initial reception, this closure regime spread. Locally, the 25 villages that now compose the ~1,000 km^2^ Velondriake LMMA oversaw 69 different octopus closures between 2004 and 2011 [48,50]. An African Development Bank project supported 50 additional closures around southwest Madagascar between 2009 and 2013 [49]. Further, beginning in 2005 the national government formalized the community initiative by shutting down the entire southwest region octopus fishery for six weeks between mid-December and late January [49]. The model also spread internationally, with the neighboring island state of Mauritius enacting similar legislation in 2012 [51]. Following the spread of the octopus closure regime, the Velondriake regional management committee took broader management steps within the LMMA, instituting periodic mangrove closures targeted at a local crab fishery, banning destructive fishing practices, engaging in ecological monitoring, and, five years after refusing the idea, instituting the first of now six permanent, community-enforced no-take areas [48,49].

The octopus fishery in Velondriake targets a group of four shallow-water species: *Octopus cyanea* (95% of local catches), *Callistoctopus macropus* (~4%), *Amphioctopus aegina* (~1%), *Callistoctopus ornatus* (rare) (D. Raberinary pers. comm, [52]). These four octopus species each have a lifecycle of about one year, dispersing as paralarvae for 2–3 months, then growing over 6–9 months from <1 g at settlement to commonly above 3 kg [53–55]. They appear to be year-round spawners, although recent studies suggest that recruitment fluctuates throughout the year [52].

The bulk of octopus is caught during spring low tides by gleaners, predominately women. They generally sell any octopus over the nationally regulated minimum size of 350g to outside buyers [56,57]. Though only 180 km north of Toliara, this region lacks transport infrastructure, rendering the isolated villages dependent on private exporting companies for market access.

Upon instituting an octopus closure, villagers typically close about one fifth of their village’s octopus harvest area (~124 ha +/- 45 CI95), for a period between 2–7 months, sometimes repeatedly (Fig 1C; Key Informant Interviews, Shawn Peabody, co-manager). The Velondriake Committee, an elected management body, selects closure sites, chooses closure durations, and coordinates management. Communities self-enforce the closures, sanctions are prescribed by local law (*dina*), and enforced by consensus at community meetings [58]. Blue Ventures Conservation, the co-managing non-governmental organization, provides technical and funding support for management efforts in cooperation with the partners mentioned above [49].

### 2.2. Socioeconomic background and surveys

Most of the approximately 7,500 people living in Velondriake (S1 and S2 Tables, S1 File) are Vezo, a subgroup of the Sakalava ethnic group whose cultural identity is tied to a fishing and gleaning lifestyle [59]. Consistent with their living in a very poor nation, Vezo populations are frequently characterized by low incomes, high resource dependence, and rapid population growth (~3% annually) [60].

To expand the local information available, a socio-economic household survey was conducted between August and September 2010 across 16 villages and 301 households (also see [61]). The 35-question household survey collected data on household demographics, income sources, fishing practices, wealth, and resource extraction habits. The survey design was based on regional socio-economic monitoring guidelines [62] and validity recommendations [63]. To ensure validity, a trained, local Vezo survey team undertook the survey in Vezo; a bi-lingual field manager supervised the teams. Pilot surveys in three villages helped inform the final survey. All survey data were double entered in Excel and quality controlled.

Villages were stratified according to geographic region (north, central, south) to account for proximity to market, and surrounding habitat (island, coast, mangrove, inland) to account for differences in fishing habits. Inland villages were eliminated from the study due to their greater dependence on farming rather than fishing and no south-island villages exist. The eight remaining strata (north-island, north-coastal, north-mangrove, central-island, central-coast, central-mangrove, south-coast, south-mangrove) allow for extrapolation to non-sampled villages. When possible, forty random households were sampled in each stratum, and female and male heads of household were alternately interviewed (S1 Table). Upon entering a village, each member of the survey team picked a random number between 1 and 12 representing the direction he or she had to walk (e.g., 3 meant 3 o’clock). Walking up to each household along that trajectory, surveyors consulted a list of previously generated random numbers between 1 and 100; if the number was below “X” then the household was sampled. “X” was different for each village, and is the (# of sampled households desired) / (the total # of households in that village).

Focus groups with fishers and gleaners in the main village of Andavadoaka provided data on market prices paid for catch, quantity and cost of gear used, and seasons (total of 12 focus groups). Interviews with fishers, commercial buyers, local middlemen (“sous collectors”), fish mongers, managers, and villagers across Velondriake (total of 26 interviews) provided information on: market prices, patterns of community decision making, local engagement with management, etc. Focus group participants and key informants were opportunistically sampled, and snowball sampling identified additional participants/informants. Market surveys and direct observations corroborated information such as market prices of fish and gear.

### 2.3. Landings Data, Sale Price, Participatory Mapping

Since 2004, trained data collectors recorded octopus landings across Velondriake at the point of sale. Each day, collectors waited at the point of sale in each participating village, which allowed easy collection of a large proportion (if not complete coverage) of the day’s catch. Collectors recorded each fishing trip including the number of fishers in the group, number of octopus caught, weight of each individual octopus, fishing site, date, village name, gear type, fisher names, fisher ages, and fisher genders. Data collected after 2008 includes octopus sex as well. The dataset’s 258,108 individual weighed octopuses from 67,990 trips were double-entered, cross-checked, and quality controlled in 2010–2011.

The price per kilogram octopus was assessed through direct observations at points of sale in June-August 2009, and trends in the “beach price” over time were confirmed through focus groups and key informant interviews (S2 Table). Prices were adjusted for inflation and purchasing power [8].

In 2009–10, we conducted participatory mapping exercises with fishers in all Velondriake’s villages to define boundaries of each fishing site. This exercise improved the managers’ and researchers’ ability to translate between local site names and specific fishing areas. A focus group of each village’s octopus fishers was asked to delineate their village’s named sites on large, laminated posters showing the satellite imagery (Google Earth) of the village’s coast. These maps were then digitized and transferred into GIS shapefiles. Each site was given a unique identifier, cross-referenced to the local site names, and confirmed with villagers [64].

### 2.4. Illegal Catch Rate

As a measure of compliance with closures, we assessed the severity of reported illegal fishing in the closed sites as “low,” “moderate,” or “high.” To assign these categories, we assessed levels of illegal fishing reported in the landings data during a closure relative to baseline catches, defined as total landings from the closure site in the 30 days before a closure. Here, “low” indicates that octopus catches recorded during the closure equaled 0–5% of baseline “before” catches; “moderate”, 5–50% of baseline; and “high” at least 50%. Fishers readily reported this activity, but nevertheless we consider these reports as a minimum estimate of illegal activity.

### 2.5. Fishery effects analysis: Landings, effort & catch per unit effort

We used a Before, After, Control, Impact (BACI) statistical design and mixed model ANOVA to test the effects of octopus closures in the Velondriake LMMA on (a) octopus fishery landings (kg octopus/30 days), (b) octopus fisher effort (total fisher-days/30 days), and (c) catch per unit effort (kg octopus/fisher-day). A subset of 36 closure events had adequate baseline data, defined as at least 5 fishers and 10 octopuses recorded in each of the 30-day periods before and after the closure (Fig 2A).

Seventeen (17) of these 36 closures occurred independently of other management measures, while the other 19 extended the six-week governmentally-imposed regional octopus fishery shutdown that was in effect in austral summer each year beginning in 2005. We refer throughout the paper to the 17 independently occurring closures as “no-ban” closures, and the 19 closures that extended the shutdown as “ban” closures (Fig 2).

#### Control Site Selection

We matched each of the 36 focal closure sites with a similar control site that (a) never had a local closure, (b) showed trends in baseline data similar to those of the closed site (i.e., the site’s monthly octopus landings; see *baseline landings correlation* (r) below), and (c) had adequate data available during the focal periods (see *relative data availability index* below). We took five steps to establish a set of impact-control pairings. (1) We prepared a baseline dataset free of ‘closure effects’ by removing data from each known closure site for the period from closure to 60 days after reopening. (2) Generate *baseline landings correlation*: To highlight site pairs that presented correlated baseline trends, we aggregated the total baseline landings of octopus by month for each fishing site, and then compared the full records of the 36 focal closed sites to 318 potential control sites using Pearson’s correlation of monthly catch totals (i.e., r). (3) Generate *relative data availability index*: To assess relative availability of data at potential control sites, we counted the minimum number of fisher-days available in either the 30 days before or after the closure, divided by the value from the control-closure pairing with the highest recorded fisher-days. Minimum available fisher-days during focal periods in selected control sites ranged from 6 to 120, with a mean of 27.6 fisher-days. (4) We then ranked each of the 11,448 potential control site-closure site pairings based on a *suitability score* that was composed of the average of the pair’s *baseline landings correlation* and *relative data availability index* during focal periods. (5) Finally, given the *suitability score* rankings, we ran a “draft-pick” algorithm, allowing each closure to select (and exclude) its top-ranked control; then randomized the selection order of the “draft-pick” set of 36 control-closure pairs over 10,000 times, taking the best global control-closure solution.

Normality and homoscedasticity of response variables were assayed using q-q plots and Levene’s test. Upon failure of either condition we log-transformed the variable in question, which met these assumptions in each case. Each analysis was performed using the lme4 package in R [65], using Period (i.e., Before or After closure), Control/Impact, and co-occurrence of the regional fishery shutdown (“ban”/”no ban”) as fixed effects, and, because multiple closure events could happen with the same closure site (at different times), we included Closure Site as a random effect. All reported significance probabilities derive from independent contrasts within this mixed-model framework [65–66].

### 2.6. Village Income

For all villages that implemented a closure event, we used a mixed model design similar to that described above to test for differences in *three variables*: (a) total village octopus income (all octopus landed in a village (kg) * beach price ($/kg); $), (b) total fishing effort (fisher-days), and (c) income per unit effort (2011 $ PPP/fisher-day) across *three periods*: (i) 30 days pre-closure (before); (ii) closure period (during; normalized to 30-day measure); (iii) 30 days post-reopening (after). We analyzed income and effort from villages implementing 28 closure events, which represent the subset of the 36 closures analyzed above for which we had data coverage to pair villages implementing a closure with control villages that had no closure at the same time. Of these 28, 14 were “no-ban” closures, and 14 were “ban” closures (Fig 2B).

### 2.7. Stochastic modeling of site-specific closure net economic benefits

To assess the site-specific net economic benefits of each closure site, we compared landings from closure sites to stochastically modeled landings assuming continued open-access fishing at the same site. To do so, we modeled both the foregone earnings for periods during and 60 days after 36 closures and then compared these modeled earnings to the actual catch data from 36 closure events (Fig 2C).

Our data provide us with two observed distributions required for our simulations: (1) **V,** the number of fishers visiting the focal site on a given day, and (2) **C**, the beach value of octopus caught by one fisher on one day. Each of these distributions are drawn directly from the fishery data, and stochastically sampled to generate our simulated data comparison (S7 Fig). To build the *visitation distribution*, **V**, we first recorded the number of fishers that went to the focal site each day it was visited during the entire study period excluding closure periods and six-weeks after a closure reopened. On days that a site received no recorded landings, there are three possibilities: (1) there was no fishing activity, (2) there was activity but no one visited the site, or (3) at least one fisher visited the site, but they caught no octopus. To accurately estimate case 2 (actual zero-visit days) we first excluded case 1 (no fishing days) by only counting zero days on which (a) there was fishing recorded in a village that had ever fished the focal site, and (b) no landings were recorded from the site in question. To further correct the estimated zero-visit days for case (3) we fit the parameter Z, where a site’s *modeled # of zero-visit days* = (Z * zero days with active fishing). We ran 100 iterations of our landings simulation model on data from 36 open-access control sites for each potential estimate of Z (from 0 to 2, by 0.025) and then calculated the difference between our modeled fishery landings and the actual landings in those 36 sites. By finding the minimum median divergence between actual and modeled values, we determined that the minimal model bias was generated with a value of Z = 0.525. All simulations thereafter used that parameter value.

This zero-bin multiplier tuning made our net economic benefit criterion more severe. That is, by roughly halving the probability of “no visit”, this provided a higher estimate of counter-factual catches (or “cost”), and therefore made our “profitability” criterion more conservative. The *value per unit effort distribution*, **C**, derives from CPUE at 150 control sites during the period in question (during or after), using beach prices on the day and village of sale.

To simulate the “no closure” catch value, for each day in which fishing is reported in a relevant village (i.e., one that has fished in the focal site in the past), we sampled from the focal site’s **V**, returning a number of fishers that visited that site that day: **V**_**d**_. Then we sampled **V**_**d**_ times from **C** to generate a distribution of single fisher’s daily catch values (**C**_**f**_). The daily sum of **C**_**f**_ over all fishers on a given day generated a time-series of total daily catch values, **L**_**d**_. The sum of daily value each day (**L**_**d**_) over all days sampled (**N**_**FD**_), generated a total landings **L**.

$$L_{d}=\sum_{f=1}^{V_{d}}C_{f}$$

$$L=\sum_{d=1}^{N_{FD}}L_{d}=\sum_{d=1}^{N_{FD}}\sum_{f=1}^{V_{d}}C_{f}$$

By treating **L**, the simulated catch value (i.e., what people would have earned had they not instituted a closure) as the *cost*, and **A**, the total actual recorded landings value from the closure site over the same time period as the *benefits*, we estimated the net earnings (**NE**) of the closure relative to its counter-factual control (**NE** = **A**-**L**). For each of the 36 modeled closures, we ran our stochastic model 1000 times resulting in a distribution of net earnings values for each closure.

### 2.8. Internal rate of return

The internal rate of return of a particular closure is the discount rate at which the net present value of the net earnings is equal to zero:

$$NPV_{NE}\;=0=\;\sum_{t=0}^{T}NE_{t}\frac{1}{{\left(1+\partial \right)}^{t}}$$

where NPV_NE_ is the net present value of the net earnings, t is the days since closure, NE_t_ is the daily net earnings, and ∂ is the discount rate. We also calculated each closure’s percentage return on investment (ROI) by dividing the net benefits by the costs.

### 2.9. Seasonality of settlement and CPUE

We assessed seasonal patterns of settlement and catch per unit effort from 2004–2011 (S9 Fig). First, to assess patterns unaffected by closure effects, we removed all closure sites from the fishery dataset. Then using an octopus’ mass at capture and a growth curve for *O*. *cyanea* [67] we back-calculated an estimate of that octopus’ settlement date. From this collection of dates, we report the relative frequency of estimated settlement events occurring on a given Julian day (S9 Fig). Then we assess the CPUE across the entire dataset on each day from 2004–2011, and present a LOESS fit of these data (with 95% confidence intervals). We then calculate the lagged cross-correlation between settlement frequency and subsequent seasonal CPUE shift.

## Results

### 3.1. Socioeconomics

The Vezo within the Velondriake LMMA have a mean income of $1.72 per person per day, below the $2 per day poverty standard, and they rely heavily on seafood protein for their food security (all $ figures presented are in 2011 international dollars, which adjusts for purchasing power parity (PPP); S1 File, S3 and S4 Tables). Gleaning contributed at least half of household income for 62% of households [S1 File], though individual fishers earn more from sea cucumbers and finfish [61].

### 3.2. Fishery landings and CPUE–BACI Analysis

**Fig 3:**
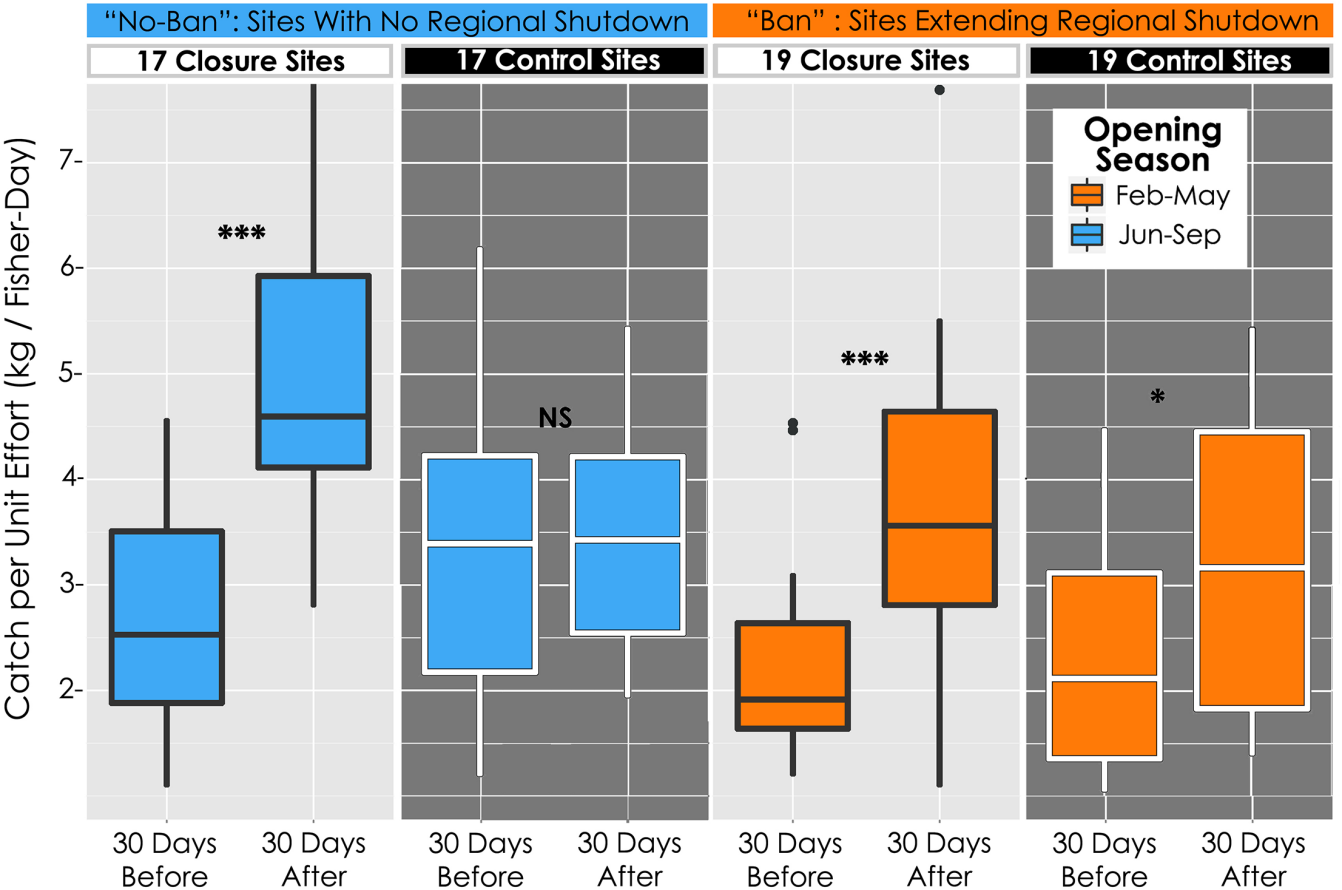
Closure effects on Catch-Per-Unit-Effort. Site-level catch-per-unit-effort (CPUE, kg/fisher-day), 30 days before closure and after reopening at closed sites and paired control sites. Data are separated by season, thus separating those closures that occurred independently of a regional fishery shutdown (“no ban”), and those that extended the shutdown (“ban”). Significance indicators show distinctions between a particular group and its “before” group comparison, independent contrasts from linear mixed models. NS = Not Significant; * = p < 0.05; *** = p < 0.001. For components of CPUE and data aggregated across seasons, please see S1 & S2 Figs.

The 36 closure sites for which we had adequate baseline data showed significant increases after re-opening in both octopus landings and CPUE of octopus, regardless of their timing with the annual regional octopus fishery shutdown (Figs 3, S1 and S2). Across the 36 closures, median octopus landings increased from 49.5 (±22.8 CI95) kg in the 30 days before closure to 404.8 (±119.9) kg in the 30 days after reopening, a 717.8% increase (p<0.0001, S2 Fig). This significant increase is robust to the timing of the regional shutdown, appearing both in the 17 “no ban” closures, that occurred independently of the regional shutdown (+550%, p<0.0001), and the 19 “ban” closures, that extended the shutdown (+821%, p<0.0001; Figs 2 and S1).

Control sites had median landings of 44.5 (±35.5) kg in the 30 days before, and 74.6 (±46.6) kg after re-opening (+67%; S2 Fig). Though this increase in control sites is statistically significant (p<0.01), it is ten-fold smaller than in closed sites (S2 and S3 Figs). Moreover, the effect in controls is driven by the 19 “ban” closures that extended the shutdown (+97%, p<0.05) and absent in the controls for the 17 “no ban” closures (+67.7%, p = 0.36; S1 Fig).

A closure’s reopening attracted many fishers. Again comparing 30-day periods immediately before each closure and after re-opening, the 36 closed sites had a median 477.8% increase in effort (fisher-days; p<0.0001, S1 and S2 Figs). There is also a weakly significant effort effect in the controls (median +74%, p = 0.05; S2 Fig), however, once split by timing relative to the regional shutdown, neither controls for the 17 “no ban” closures nor those for the 19 “ban” closures showed significant effort increases (median +88%, p = 0.37; +117%, p = 0.28; Figs 2, 3 and S1).

Catch per unit effort (kg/fisher-day, CPUE) showed large and significant increases at closure sites while control sites showed only a minor boost, which was again restricted to those “ban” closures co-occurring with the regional shutdown (Figs 2 and S2). In closure sites fishers caught a median of 2.37 (±0.33) kg of octopus per fisher-day before closure, but after the closure re-opened, fishers caught 4.42 (±0.51) kg/fisher-day, a CPUE increase of 86.6% (p<<0.0001; S2 Fig). These significant CPUE increases were present both in “ban” and “no ban” closures (Figs 2 and 3). In control sites for the 17 “no ban” closures, median CPUE showed no significant change (p = 0.93; Fig 3), while controls for the 19 “ban” closures showed a moderate boost (+49%, p<0.01; Fig 3).

Both the landings and CPUE boosts were greatest immediately after the closure’s reopening, and diminished within days to weeks after the opening (S3 and S4 Figs). Landings tended to return to baseline levels after the first or second tidal series, generally within about 7–10 days after reopening (S3 Fig). CPUE effects were also strongest in the first set of spring low tides after the opening, but continued into the second or third set of spring low tides (i.e., 14–25 days; S4 Fig). As this fishery is depth limited, most active fishing occurs during the lowest tides (i.e. spring) tides.

### 3.2. Village-level fishery income

**Fig 4:**
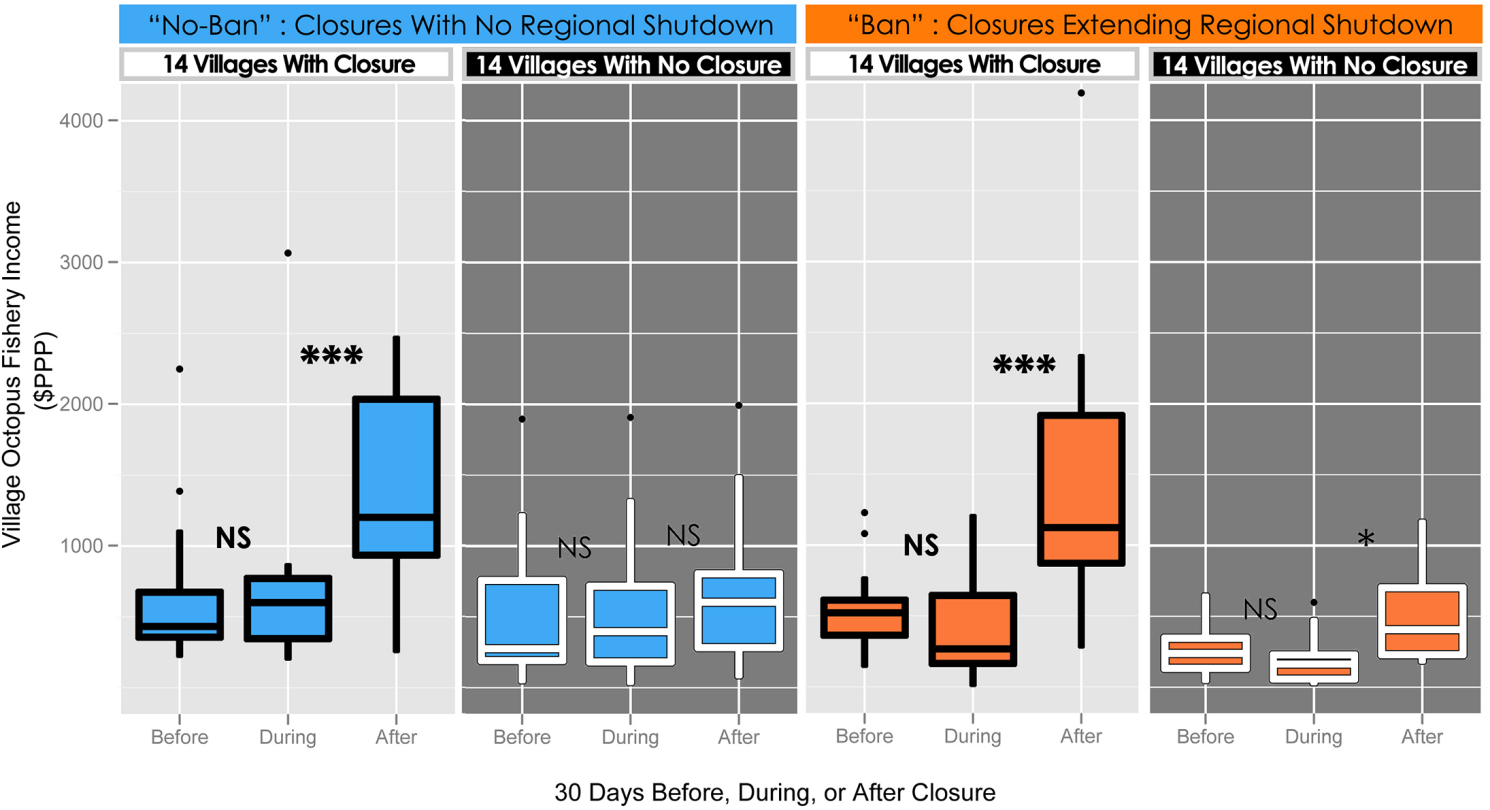
Closure effects on Village Fishery Income. Total village-level octopus fishing income ($PPP) 30 days before, during, and after closures, at villages both with and without closures. The data depicted are from 28 closure periods showing closure-implementing villages and their control villages from 2004–2011. Data are separated by season, thus separating closures that occurred independently of a regional fishery shutdown (“no ban”), and those that extended the shutdown (“ban”). As “during” periods are not exactly 30 days, “during” values are scaled to a per-30-day measure. Significance indicators show distinctions between a particular group and its “before” group comparison, from linear mixed-effect model. NS = Not Significant; * = p < 0.05; ** = p < 0.01; *** = p < 0.001. For effort, value per unit effort, and data aggregated across seasons, please see S5 & S6 Figs.

After closures reopened, villages that implemented closures experienced higher incomes from octopus fisheries compared to villages within the LMMA that did not implement closures (Fig 4, Table 1). In the 28 examined closure events, the implementing villages had mean octopus-fishery income of $597 (±$168) for the 30 days before the closure, and $1,407 (±$322) in the 30 days after the closure reopened, an increase of 136% (p < 1e-5; Figs 2 and 4, S5 Fig). While villages saw significant positive net benefits from closures, their apparent costs due to foregone catch were not statistically distinguishable. On average, neither implementing nor control villages experienced a consistent, significant decline in octopus income during the closure periods relative to the 30 days before (p = NS; Figs 4, S5 and S6). For villages with closed sites, both the significant income increase after reopening and the lack of income decline during the closure were robust to co-occurrence with the regional shutdown (i.e., “ban”/“no ban”; Figs 2, 4 and S6). Conversely, income effects in control villages (with no closed sites) depended on the timing of the regional octopus fishery shutdown. For “no ban” closures, control villages had no significant change in village-level octopus fishery income before, during, or after the closures (p = 0.25; Fig 4). However, for those “ban” closures that extended the regional fishery shutdown, there was a weakly significant income increase in control villages comparing before to after periods (+88%, p = 0.043; Fig 4).

**Table 1:** Mean Village Level Octopus Income Before and After Temporary Closures.

	Foregone Catch $PPP During-Before	Opening Boost $PPP After-Before	Effort Change During-Before	Effort Change After-Before	Income per Effort During-Before	Income per Effort After-Before	% Fished Area Closed
**ALL CLOSURES**							
Closure Villages (36)	-$1	$817	-14.8%	102.5%	$0.54	$1.36	18.6%
Control Villages (28)	-$17	$214	-7.9%	95.4%	$0.37	$0.66	0.0%
**CLOSURES INDEPENDENT OF REGIONAL SHUTDOWN–“NO BAN”**							
Closure Villages (17)	$104	$865	5.3%	100.3%	$0.55	$1.48	16.6%
Control Villages (14)	$49	$189	12.7%	58.5%	$0.64	$1.02	0.0%
**CLOSURES EXTENDING REGIONAL SHUTDOWN–“BAN”**							
Closure Villages (19)	-$96	$775	-32.8%	104.5%	$0.53	$1.25	20.4%
Control Villages (14)	-$82	$240	-28.5%	132.3%	$0.09	$0.30	0.0%

### 3.3. Site-level net economic benefits, internal rate of return, and return on investment

**Fig 5:**
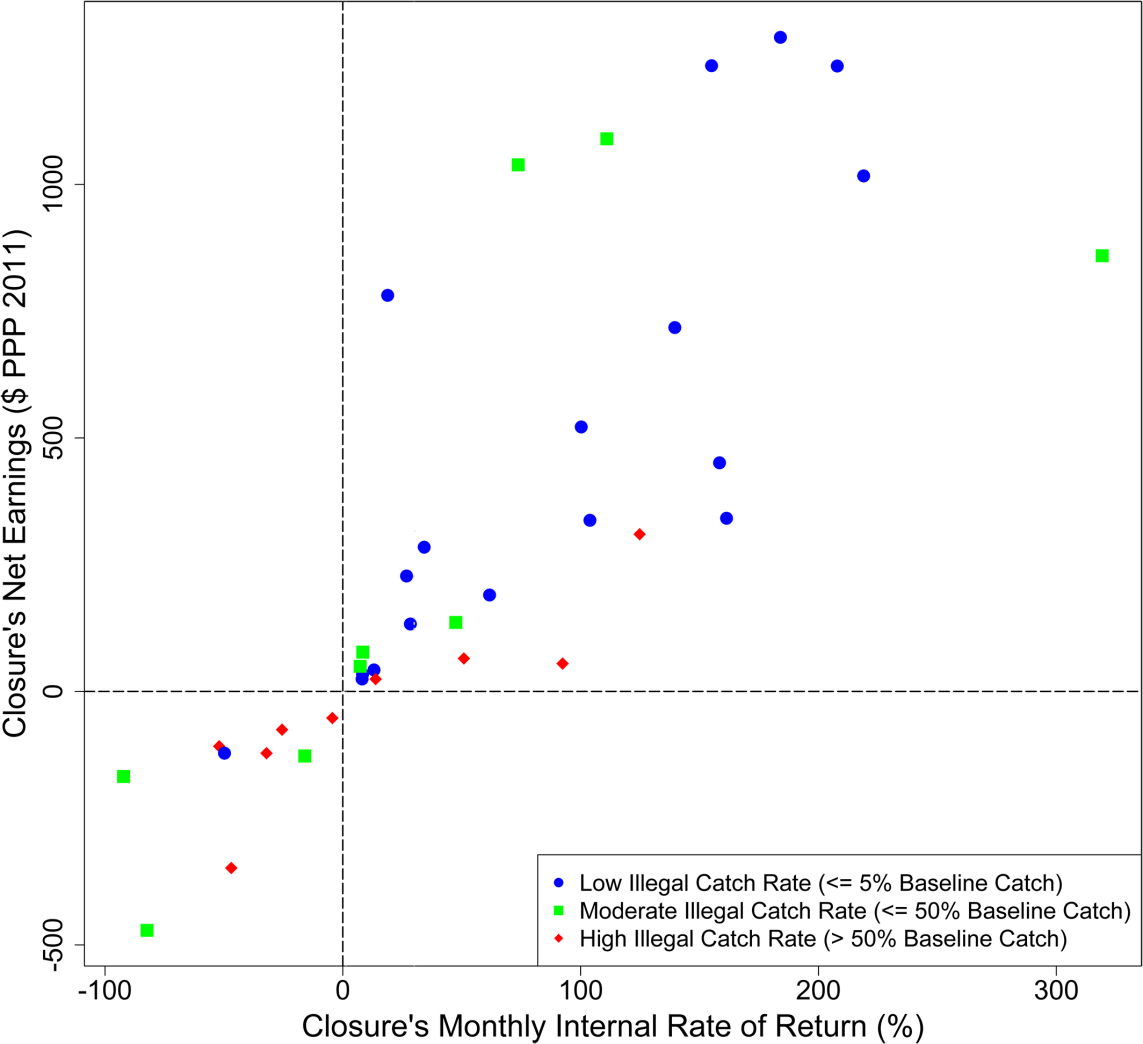
Net Economic Benefits of Closures. Site-specific Net Earnings (NE) and Internal Rate of Return (IRR) of 36 local closures, 2004–2011, using site-based cost model. Point coding represents rates of illegal fishing during the closures: Low (< = 5% of baseline ‘before’ catches, blue circles), Moderate (< = 50% of baseline catches, green squares), and High (>50% of baseline catches, red diamonds). Data points represent median values across 1000 model runs.

We present the un-discounted net earnings (NE), the monthly internal rate of return (IRR), and the percentage return on investment (ROI) for each site (Figs 5 and S8). Each of these values represents the median value after 1,000 model runs for each of the 36 closures.

The majority (27 of 36; 75.0%) of the closures were strictly profitable, with positive NE, monthly IRRs, and ROIs (Table 2; Fig 5). The 36 closures netted the 12 implementing villages a mean of $305 per closure (Table 2). The majority of closures also showed rapid returns on investment. The monthly IRRs of the 36 closures range from negative (a loss) to 319%, with a mean value of 58% (+/- 30% CI95; Table 2, Fig 5), suggesting that the closures would satisfy someone expecting half of their investment returned in a month, i.e., someone having a monthly discount rate of 50%. The mean ROI of the 36 closures was 81% (+/- 42% CI95), implying that, on average, one dollar’s worth of octopus left in the ocean grew to $1.81 by the end of a closure.

**Table 2:** Closure Site Net Economic Benefits.

	Foregone Catch ($ PPP)	Benefit ($ PPP)	NE ($ PPP)	Monthly IRR (%)	ROI (%)
**All Closures (N = 36)**					
**Total**	-$18,294	$29,270	$10,976		
**Mean**	-$508	$813	$305	57.7%	80.9%
**95%CI**	$105	$193	$156	30.3%	42.0%
**Closures Independent of Regional Shutdown (“No Ban” N = 17)**					
**Total**	-$9,834	$15,684	$5,850		
**Mean**	-$578	$923	$344	84.7%	90.7%
**95%CI**	$173	$297	$239	49.9%	51.3%
**Closures Extending Regional Shutdown (“Ban” N = 19)**					
**Total**	-$8,460	$13,586	$5,126		
**Mean**	-$445	$715	$270	33.5%	72.1%
**95%CI**	$122	$251	$210	33.7%	66.1%

NE and IRR declined as illegal catches increased (Figs 5 and S8). This NE decline showed most tangibly in lowered post-opening landings (i.e., benefits) and less so in costs due to foregone catch. Conversely, in those sites with little reported illegal catch, we see a consistent pattern of positive earnings and rapid rates of return (Figs 5 and S8, Table 3).

**Table 3:** Effect of Illegal Fishing on Mean Closure Site Net Economic Benefits.

Level of Illegal Fishing	#	NE	IRR	ROI
Low	18	$486 (+/- $211)	88% (+/- 37%)	123% (+/- 62%)
Moderate	9	$276 (+/- $373)	42% (+/- 80%)	66% (+/- 92%)
High	9	$-28 (+/- $116)	13% (+/- 41%)	11% (+/- 43%)

### 3.4. Seasonality

We examined seasonal patterns in closure effects (Tables 1 and 2), CPUE, and larval settlement (back-calculated from growth-curves). Closures show stronger positive effects on CPUE and village incomes during the austral winter (i.e., in “no ban” closures) than in austral summer (i.e., “ban” closures; Tables 1 and 2). Across the entire fishery dataset with closure sites removed, CPUE also shows clear seasonal trends with maximal CPUE occurring in austral winter (S9 Fig). Using lagged cross-correlations between Loess-fitted CPUE and larval settlement indices (maximal between 4.5–5.5 months), we show that correlations with the settlement trends can account for about 26% of the variation in the seasonal CPUE trend (r = 0.51, r^2^ = 0.26; S9 Fig).

## Discussion

### 4.1. Results summary

Periodic closures in the Velondriake LMMA’s octopus fishery had positive impacts. Both octopus landings and CPUE increased significantly above baseline levels upon re-opening of closed areas (Fig 3). Villages implementing closures saw a doubling of octopus fishery income after reopening, and saw no significant decline of income during closures (Fig 4). This lack of income decline during closures suggests that fishers displaced their effort from the roughly 20% of a village’s fished area that was closed to the remaining open-access areas fished by their villages. Closures also showed positive net economic benefits at the site level using a conservative cost model that does not allow income from displaced effort to offset costs from foregone catch. These benefits were dependent upon good enforcement, as higher rates of illegal catch from the closure site eroded net earnings (Figs 5 and S8; Table 3).

Both site and village level effects were absent in the open-access controls for the 17 “no-ban” closures that occurred independently of other management measures. However, open-access controls for the 19 “ban” closures, during which all sites in the region were shut-down for the first six weeks of a closure’s 2–3 month duration, showed significant if comparatively small effects in landings, CPUE, and village income. While these “ban” effects partially confound the local closure impacts we were trying to measure, the fact that they also showed positive results provides further evidence that short-term closure regimes targeting rapidly growing organisms can generate fishery and economic benefits.

### 4.2. Net income benefits in local context: Are income boosts meaningful?

As Velondriake’s periodic closure regime generated tangible benefits and little to no foregone catches, the increased net earnings attributable to the closures provided non-trivial welfare gains. Overall, each of the 36 closures resulted in a mean boost in benefits of $817 per implementing village(s) (Table 1), more than doubling (+136%) the baseline income from the octopus fishery over the focal 30 days. Translating these gains into the effect on a village’s daily life, this boost over baseline implies a mean benefit per unit effort (fisher-day) of $2.36. This translates to an extra $2.36–4.72 per household per day fished in a context where the average household earns just $7.77 per day [S1 File]. For each day spent gleaning octopus in this post-opening period this income boost alone would supply the household with an extra 1–2 days of rice or 2.5–5 days of fish.

### 4.3. Local time preferences: Do returns accrue fast enough to satisfy subsistence fishers?

For the income gains we report to be perceived as economically beneficial to local fishers, they must have accrued at rates fast enough to satisfy local preferences. Twenty-seven of the 36 examined closures produced positive net earnings (NE), but for an action to be perceived as economically “worth the effort”, high NE must be paired with internal rates of return (IRRs) high enough to match local time preferences [68].

In economic terms, time preference is the relative value of immediate versus delayed rewards, and is expressed as the "discount rate" [68]. Individual time preferences are notoriously difficult to measure and compare, in part because they are very sensitive to elicitation or observation method [69]. Estimates of Vezo discount rates span a wide range. In assessing development projects, practitioners often apply a rate of 10–12% annually in the developing world (0.8–0.95% monthly). A discrete choice experiment designed to assess long-term ecosystem service values within Velondriake found fishers’ discount rates around 4.1% monthly [70], substantially lower than measures from other subsistence fishers who routinely show rates above 10% monthly [71]. An anthropological study targeting Vezo at the southern border of Velondriake found higher rates in which the modal response fell between 30% and 47% monthly [72].

Using the standard development project rate (0.8–0.95% monthly) or that estimated from within Velondriake (4.1% monthly) all closures with positive NE (27 of 36) generated returns rapidly enough to satisfy Vezo fishers (Fig 5). Even when using the higher estimated range (30–47% monthly), over 60% of closures with positive NE did not disappoint their sponsors: 17 of the 27 “profitable” closures had IRRs above 47% (and three more were close, at 27%, 28%, and 34%). This site-based model’s economic benefit outcomes are conservative, because in practice, as we show in the village-level analysis, fishers were able to divert fishing effort to unmanaged sites during closure periods and thereby catch octopuses that were not accounted for in this site-focused theoretical model. What the economic results do suggest is that 75% of closures met a strict “profitability” criterion and between 60–100% of those “profitable” closures produced net economic benefits rapidly enough to satisfy extremely high discount rates.

Understanding resource users’ discount rates, and how they affect behavior [73], can guide managers’ choice of appropriate management tools, as local discount rates are intimately tied to resource extraction and environmental stewardship [74,75]. In places where discount rates are high, the time horizon of management’s pay-off is critical to community acceptance.

### 4.4. Local management relevance: enforcement & seasonality

In village meetings, communities spent considerable time discussing the details of proposed closed sites, such as duration, size, enforceability, and timing (Key Informant Interview, Shawn Peabody, co-manager). For the closures in this study, neither closure size nor duration had significant effects on net economic benefits, but both effective enforcement and seasonal timing did.

Without effective enforcement, there is no reliable post-closure benefit and the most economically rewarding strategy reverts to open-access fishing. As the closure regime spread, unenforced, or broken, closures occurred from time to time (Key Informant Interview, Roger Samba, community leader). Fishers reported catches within closed areas to the buyers and data collectors because these agents had no enforcement or sanctioning duty, or in some cases villages chose not to enforce a planned closure. These reported illegal catches nonetheless likely represent a minimum estimate of illegal catch. As effective enforcement had the clearest impacts on a closure’s net earnings, strategies focusing on improving effective enforcement of the closed area would likely return tangible results.

Seasonality may also be an important consideration. Other higher latitude octopus fisheries show patterns of strong seasonality [76–78], but seasonal patterns in maturity appear comparatively more subtle in the Malagasy stocks [52]. Nonetheless, the village and site-level analyses present evidence that closures showed larger effects during the austral winter season (Jun-Sep), as opposed to those that extended the regional shutdown and opened in summer (Feb; Tables 1 and 2). This profitability pattern matches the seasonal fluctuation in CPUE across the entire fishery (with closure effects removed), which peaks in June-August (S9 Fig). These annual patterns in CPUE and stronger closure effects might be explained by a broad peak in estimated *O*. *cyanea* larval settlement that occurs in February-March, with a narrower peak in September. Closures set in the austral winter (Jun-Aug) may protect the settlers from earlier in the year (Jan-Mar), during a period of near-maximum growth rates [67]. Although explicit population models of settlement and growth are beyond the scope of this paper, this remains an enticing, if underexplored hypothesis.

### 4.5. Transferability of the approach

In any setting, the optimal periodic closure regime must be designed around the specific bio-economics and governance context. Further examining this case can provide insight into the transferability of the approach and results, as well as lessons, for other locations. This case is an example of a successful periodic harvest regime that focused on a particularly appropriate single species (Octopus) and fishery (low selectivity, low harvesting cost, traditional gear), in a context that had some, but not all, of the factors thought to facilitate success [13,16,23,24,29,46,52].

First, biologically, octopus may have been an ideal species to manage. Models show that rapid growth and short lifespans allow for shorter optimal closure times [31,32,43], and *O*. *cyanea* grows rapidly and non-asymptotically, completing its lifecycle within a year [55,67,78].

Second, the nature of the fishery may have been a critical factor in the success of the periodic closures. Poor selectivity of size classes in a fishery and high intensity harvesting skew the optimal harvest regime from stationary to periodic harvests [31,43]; the Velondriake octopus fishery was not selective enough to avoid juvenile capture, both due to the style of fishing (blind spearing into a den [79]) and because year-round spawning and settlement reduce seasonal, age-homogeneous cohorts [52]. Low harvesting costs also can lead to high intensity harvesting; gear for the Velondriake fishery was generally a spear and a bucket, and in rare cases a mask [61], and everyone fished for octopus on a regular basis [61].

Third, the economics of the fishery were important. Again, bio-economic models suggest that the value of the landings and discount rate are key considerations [43]. In Velondriake, the product generated significant cash for the communities [61], and the wealth was distributed relatively evenly across the community thanks to broad participation and minimal access restrictions [61,80,81]. Moreover, the reward for management was accrued rapidly, at a rate that satisfied Vezo fishers’ discount rates.

Finally, experience suggests governance and social factors are critical to successfully constrain fishing patterns to the optimal schedule [22–24,29,45]. Velondriake had strong leadership that people trusted and high levels of social capital [82]. Velondriake did not possess all social factors typically cited as critical for success, however. According to our household survey and previous surveys, local knowledge of the human impact on ecological systems was weak. And, while some periodic closure studies suggest that exclusive tenure increases the likelihood of success of periodic closures, our case was more in line with the body of empirical evidence that common property institutions can successfully implement management without property rights over the resource [17].

### 4.6. A Community management catalyst?

This case adds to a growing body of evidence of a pattern where experience with effective periodic closures leads to broader management. Following the wide adoption of this closure regime, the communities within the Velondriake area adopted a substantially broader range of community-based and co-management actions. Such actions included: the formation of an LMMA represented by a governing body, the Velondriake Committee [49]; the extension of the periodic closure regime into mangrove habitats [49]; the banning of destructive fishing methods [58]; the formal gazetting of the Velondriake LMMA [48] and finally, the founding and community enforcement of six no-take marine reserves [48]. This pattern mirrors experiences with periodic closures in Vanuatu and Indonesia, where support for the limited closure regimes facilitated community engagement in broader management [45,47].

This pattern also appears to be a plausible mechanism when examined in light of the literature on successful management of common pool resources [13,16,17,46]. A low cost and economically effective periodic harvest regime passes a fundamental principle from common pool resource theory: that the local fisher community perceives that expected management benefits outweigh the costs of organizing [46]. Once in place, the activities associated with managing a multi-village periodic closure regime may positively affect an important subset of criteria the commons literature has found to be crucial for management self-organization: the potential for local leadership to arise [13,46]; an increase in inter-village communication and building of social capital and trust [13,16,46]; improved knowledge of humans’ effects on the resource system [16,46]; and the ability to craft and enforce collective choice rules [13,46]. By building better conditions for cooperation [46], the management of an effective periodic closure regime may lower the metaphorical activation energy for other, broader management, just as a catalyst would in a chemical reaction. This potential is by no means a panacea for community management ills [83], but suggests that targeted and effective management might help catalyze broader community management efforts. Further study of this apparent pattern and the catalyst hypothesis could reveal important lessons for achieving desired ecological, social, and governance outcomes in small-scale fisheries contexts across the world.

## Conclusions

Periodic, temporary fishery closures targeted at rapidly growing species can have positive economic benefits for low income fishing communities and can be a promising option for the coastal management portfolio in less developed nations. Analysis of one regime in southwest Madagascar suggests that the returns are substantial, rapid, and recurring. The short history of management in the region also suggests that short-term interventions that demonstrate tangible management benefits may aid in the development of broader community and co-management efforts. Formal studies of this “community catalyst hypothesis” would greatly clarify the potential utility of periodic closures as part of a broader community-based management portfolio.

## Supporting Information

S1 Fig Seasonal Fishery Impacts. Site-level (a) octopus landings (kg) and (b) fishing effort (fisher-days), 30 days before and after closure at closed sites and paired control sites. Data are separated by season, thus separating those closures that occurred independently of a regional fishery shutdown (“no ban”, 17/36), and those that extended the shutdown (“ban”, 19/36). Significance indicators show distinctions between a particular group and its “before” group comparison, independent contrasts from linear mixed models. NS = Not Significant; * = p<0.05; *** = p < 0.001. Seasons indicate timing of closure reopening, Spring = February-May, Fall = June-September.
(JPG)

S2 Fig Aggregate Fishery Impacts. Site-level (a) octopus landings (kg), (b) fishing effort (fisher-days), and (c) catch-per-unit effort (kg/fisher-day), 30 days before and after closure at closed sites and paired control sites. Data are aggregated across season/fishery shutdown. Significance indicators show distinctions between a particular group and its “before” group comparison, independent contrasts from linear mixed models. NS = Not Significant; * = p<0.05; ** = p<0.01; *** = p < 0.001.
(JPG)

S3 Fig Daily Landings. Site-level octopus landings (kg) 30 days before and after closure at closed sites (upper, red) and paired control sites (lower, blue). Data are separated by season, thus separating those closures that occurred independently of a regional fishery shutdown (“no ban”, 17/36), and those that extended the shutdown (“ban”, 19/36). Raw daily totals are shown as gray points, with an overall mean and 95% CI shown as a red/blue line ‘before’, and a LOESS fit with 95%CI shown ‘after’. Note that due to timing the opening with the low spring tide, data ‘after’ are in phase with tidal cycles, while those ‘before’ are not.
(JPG)

S4 Fig Daily CPUE. Site-level catch-per-unit-effort (kg/fisher-day) 30 days before and after closure at closed sites (upper, red) and paired control sites (lower, blue). Data are separated by season, thus separating those closures that occurred independently of a regional fishery shutdown (“no ban”, 17/36), and those that extended the shutdown (“ban”, 19/36). Raw daily totals are shown as gray points, with an overall mean and 95% CI shown as a red/blue line ‘before’, and a LOESS fit with 95%CI shown ‘after’. Note that due to timing the opening with the low spring tide, data ‘after’ are in phase with tidal cycles, while those ‘before’ are not.
(JPG)

S5 Fig Village Income. Total village-level octopus fishing income ($PPP), and village-level octopus fishing income per unit of fishing effort ($PPP/fisher-day) 30 days before, during, and after closures, at villages both with and without closures. The data depicted are from 28 closure periods showing closure-sponsoring villages and their paired control villages from 2004–2011. Data are aggregated by season. As “during” periods are not exactly 30 days, “during” values are scaled to a per-30-day measure. Significance indicators show distinctions between a particular group and its “before” group comparison, from linear mixed-effect model. NS = Not Significant; * = p < 0.05; ** = p < 0.01; *** = p < 0.001.
(JPG)

S6 Fig Village Effort, VPUE. Total village-level octopus fishing effort (fisher-days) and village-level octopus fishing income per unit effort ($PPP/fisher-day) 30 days before, during, and after closures, at villages both with and without closures. The data depicted are from 28 closure periods showing closure-sponsoring villages and their paired control villages from 2004–2011. Data are separated by season, thus separating those closures that extended a regional fishery shutdown, and those that occurred independently of the shutdown. As “during” periods are not exactly 30 days, “during” values are scaled to a per-30-day measure. Significance indicators show distinctions between a particular group and its “before” group comparison, from linear mixed-effect model. NS = Not Significant; * = p < 0.05; ** = p < 0.01; *** = p < 0.001.
(JPG)

S7 Fig Site-base model example. Here we plot an example of a single site in our site-based cost model, showing a timeline of the closure, with closure period (blue hatching), presence of fishing in nearby villages (x’s along bottom), actual recorded landings (i.e. closure ‘benefits’, black circles/bars), modeled foregone landings (i.e. closure ‘costs’, red circles/bars), and running sums (gray bars). Below we see the three distributions from which the counter-factual landings are simulated: the pattern of visitation at this site across the whole dataset (excluding data from closure and post-closure periods), and value-per-unit effort distributions both during and 60-days after the closure (again, excluding data from closure and post-closure periods).
(JPG)

S8 Fig Illegal Fishing Rate vs. NE & IRR. Here we compare a site’s modeled Net Earnings and monthly IRR to Illegal Catch Rates expressed as reported landings from a closed site during the closure as a percentage of baseline landings during the 60 days before closure. Comparisons among both NE & IRR, and Illegal catch rate are shown both as continuous regressions, and across catch rate categories.
(JPG)

S9 Fig Seasonality in Settlement and Catch-Per-Unit-Effort (CPUE). Here we plot frequency of octopus settlement (as back-calculated from individual mass at capture and a species-level growth curve) with Julian day, including data from 2004–2011 (blue bars). Overlaid on these data is a LOESS fit of seasonal CPUE variation with 95%CI (red lines), with closure sites removed. In green we show the timing of the annual regional fishery shutdown, which began in 2006. Note the increase in CPUE variability during this more data-poor time. Below we plot the cross-correlation of these trends, showing a maximal correlation at 159 days.
(PNG)

S1 File Description of supplementary socioeconomic results. (DOCX)

S1 Table Estimated population and number of households and household survey sample size by stratum. (DOCX)

S2 Table Village characteristics: stratum, population, and households. (DOCX)

S3 Table Average household incomes across regions and habitats (2011 MGA per week). (DOCX)

S4 Table Average daily per capita income across regions and habitats (2011 PPP per person per day). (DOCX)

S5 Table Fishers’ octopus sale prices. (DOCX)

## References

[1] Kent G (1997) Fisheries, food security, and the poor. Food Policy 22: 393–404. Available: http://www.sciencedirect.com/science/article/pii/S0306919297000304.

[2] Cesar H, Burke L, Pet-Soede L (2003) The Economics of Worldwide Coral Reef Degradation. Cesar Environmental Economics Consulting (CEEC). Available: http://www.panda.org/coral.

[3] Allison EH, Perry AL, Badjeck M-C, Adger WN, Brown K, Conway D, et al (2009) Vulnerability of national economies to the impacts of climate change on fisheries. Fish and fisheries 10: 173–196. 10.1111/j.1467-2979.2008.00310.x

[4] Burke LM, Reytar K, Spalding M, Perry A (2011) Reefs at risk revisited World Resources Institute Washington, DC.

[5] Andréfouët S, Muller-Karger FE, Robinson JA, Kranenburg CJ, Torres-Pulliza D, Spraggins SA, et al. (2006) Global assessment of modern coral reef extent and diversity for regional science and management applications: a view from space. Proceedings of 10th International Coral Reef Symposium: 1732–1745.

[6] Acheson JM (2006) Institutional failure in resource management. Annual Review of Anthropology 35: 117–134. 10.1146/annurev.anthro.35.081705.123238

[7] Allison EH (2001) Big laws, small catches: global ocean governance and the fisheries crisis. J Int Dev 13: 933–950. 10.1002/jid.834

[8] World Bank (2013) World Development Indicators. Available: http://data.worldbank.org/data-catalog/world-development-indicators.

[9] Evans L, Cherrett N, Pemsl D (2011) Assessing the impact of fisheries co-management interventions in developing countries: A meta-analysis. J Environ Manage 92: 1938–1949. 10.1016/j.jenvman.2011.03.010 21531068

[10] Allison G, Lubchenco J, Carr M (1998) Marine reserves are necessary but not sufficient for marine conservation. Ecol Appl 8: 79–92. Available: http://www.esajournals.org/doi/abs/10.1890/1051-0761(1998)8%5BS79:MRANBN%5D2.0.CO%3B2.

[11] Wamukota AW, Cinner JE, McClanahan TR (2012) Co-management of coral reef fisheries: a critical evaluation of the literature. Marine Policy 36: 481–488.

[12] Pollnac R, Crawford B, Gorospe M (2001) Discovering factors that influence the success of community-based marine protected areas in the Visayas, Philippines. Ocean Coast Manag 44: 683–710.

[13] Gutiérrez NL, Hilborn R, Defeo O (2011) Leadership, social capital and incentives promote successful fisheries. Nature 470: 386–389. 10.1038/nature09689 21209616

[14] Johannes RE (2002) The Renaissance of Community-Based Marine Resource Management in Oceania. Annu Rev Ecol Syst 33: 317–340. 10.1146/annurev.ecolsys.33.010802.150524

[15] Cinner JE, Daw TM, McClanahan TR, Muthiga N, Abunge C, Hamed S, et al (2012) Transitions toward co-management: The process of marine resource management devolution in three east African countries. Glob Environ Change 22: 651–658.

[16] Cinner JE, McClanahan TR, Macneil MA, Graham NAJ, Daw TM, Mukminin A, et al (2012) Comanagement of coral reef social-ecological systems. Proc Natl Acad Sci U S A 109: 5219–5222. 10.1073/pnas.1121215109 22431631 PMC3325732

[17] Ostrom E (1990) Governing the Commons Cambridge University Press. 1 pp.

[18] Berkes F (2006) From community-based resource management to complex systems: the scale issue and marine commons. Ecol Soc 11: 45.

[19] Barrett CB, Travis AJ, Dasgupta P (2011) Biodiversity Conservation and Poverty Traps Special Feature: From the Cover: On biodiversity conservation and poverty traps. Proc Natl Acad Sci U S A 108: 13907–13912. 10.1073/pnas.1011521108 21873176 PMC3161563

[20] Johannes R (1978) Traditional marine conservation methods in Oceania and their demise. Annu Rev Ecol Syst 9: 349–336. Available: http://www.jstor.org/stable/10.2307/2096753.

[21] Jokiel PL, Rodgers KS, WALSH WJ, Polhemus DA, Wilhelm TA (2011) Marine Resource Management in the Hawaiian Archipelago: The Traditional Hawaiian System in Relation to the Western Approach. J Mar Biol 2011: 1–16. 10.1007/s00338-003-0317-2

[22] Bartlett CY, Manua C, Cinner J, Sutton S, Jimmy R, South R, et al (2009) Comparison of outcomes of permanently closed and periodically harvested coral reef reserves. Conserv Biol 23: 1475–1484. 10.1111/j.1523-1739.2009.01293.x 19624531

[23] Cinner J, Marnane MJ, McClanahan TR, Almany GR (2006) Periodic closures as adaptive coral reef management in the Indo-Pacific. Ecol Soc 11: 31.

[24] Cohen PJ, Foale SJ (2013) Sustaining small-scale fisheries with periodically harvested marine reserves. Marine Policy 37: 278–287.

[25] Johannes RE (1982) Traditional conservation methods and protected marine areas in Oceania. Ambio.

[26] Ruttan LM (1998) Closing the commons: Cooperation for gain or restraint? Hum Ecol 26: 43–66.

[27] Hviding E (1996) Guardians of Marovo Lagoon: Practice, Place, and Politics in Maritime Melanesia Honolulu, HI USA: University of Hawaii Press. 1 pp.

[28] Jupiter SD, Weeks R, Jenkins AP, Egli DP, Cakacaka A (2012) Effects of a single intensive harvest event on fish populations inside a customary marine closure. Coral Reefs 31: 321–334.

[29] Da Rocha J-M, Gutiérrez M-J, Antelo LT (2012) Pulse vs. optimal stationary fishing: The Northern Stock of Hake. Fish Res 121–122: 51–62. 10.1016/j.fishres.2012.01.009

[30] Clark C, Edwards G, Friedlaender M (1973) Beverton-Holt Model of a Commercial Fishery: Optimal Dynamics. J Fish Res Bd Can 30: 1629–1640. 10.1139/f73-262

[31] Hannesson R (1975) Fishery Dynamics: A North Atlantic Cod Fishery. Can J Econ 8: 151–173.

[32] Caddy JF, Seijo JC (1998) Application of a spatial model to explore rotating harvest strategies for sedentary species. In: Jamieson GS, Campbell A, editors. Proceedings of the North Pacific Symposium on Invertebrate Stock Assessment. Canadian Special Publication of Fisheries and Aquatic Sciences. pp. 359–366.

[33] Pfister CA, Bradbury A (1996) Harvesting Red Sea Urchins: Recent Effects and Future Predictions. Ecol Appl 6: 298 10.2307/2269573

[34] Valderrama D, Anderson JL (2007) Improving Utilization of the Atlantic Sea Scallop Resource: An Analysis of Rotational Management of Fishing Grounds. Land Econ 83: 86–103.

[35] Hart DR (2003) Yield-and biomass-per-recruit analysis for rotational fisheries, with an application to the Atlantic sea scallop (Placopecten magellanicus). Fish Bull (Wash D C) 101: 44–57.

[36] Sluczanowski PR (1984) A Management Oriented Model of an Abalone Fishery Whose Substocks are Subject to Pulse Fishing. Can J Fish Aquat Sci 41: 1008–1014. 10.1139/f84-117

[37] Nash W, Adams T, Tuara P, Terekia O, Munro D (1995) The Aitutaki trochus fishery: a case study. South Pacific Commission. 1 pp.

[38] Foale S (1998) Assessment and management of the trochus fishery at West Nggela, Solomon Islands: an interdisciplinary approach. Ocean Coast Manag 40: 187–205.

[39] Cohen PJ, Alexander TJ (2013) Catch rates, composition and fish size from reefs managed with periodically-harvested closures. PLoS ONE 8: e73383 10.1371/journal.pone.0073383 24066044 PMC3774770

[40] Williams ID, Walsh L, Miyasaka A, Friedlander AM (2006) Effects of rotational closure on coral reef fishes in Waikiki-Diamond head fishery management area, Oahu, Hawaii. Mar Ecol Prog Ser 310: 139–149.

[41] Cohen PJ, Cinner JE, Foale S (2013) Fishing dynamics associated with periodically harvested marine closures. Glob Environ Change 23: 1702–1713. 10.1016/j.gloenvcha.2013.08.010

[42] Clark CW (2010) Mathematical bioeconomics: the mathematics of conservation 3rd ed. Hoboken, New Jersey: John Wiley & Sons. 1 pp.

[43] Da Rocha J-M, Gutiérrez M-J, Antelo LT (2012) Selectivity, Pulse Fishing and Endogenous Lifespan in Beverton-Holt Models. Environ Resour Econ (Dordr) 54: 139–154. 10.1007/s10640-012-9585-z

[44] Diekert FK, Hjermann DØ, Nævdal E, Stenseth NC (2010) Non-cooperative exploitation of multi-cohort fisheries—The role of gear selectivity in the North-East Arctic cod fishery. Resour Energy Econ 32: 78–92. 10.1016/j.reseneeco.2009.09.002

[45] Harkes I, Novaczek I (2002) Presence, performance, and institutional resilience of sasi, a traditional management institution in Central Maluku, Indonesia. Ocean Coast Manag 45: 237–260.

[46] Ostrom E (2009) A general framework for analyzing sustainability of social-ecological systems. Science 325: 419–422. 10.1126/science.1172133 19628857

[47] Johannes RE (1998) Government-supported, village-based management of marine resources in Vanuatu. Ocean Coast Manag 40: 165–186. 10.1016/S0964-5691(98)00046-5

[48] Harris AR (2011) Out of sight but no longer out of mind: a climate of change for marine conservation in Madagascar. Madag Conserv Dev 6 10.4314/mcd.v6i1.68058

[49] Harris A (2007) “To live with the Sea” Development of the Velondriake Community-Managed Protected Area Network, Southwest Madagascar. Madag Conserv Dev 2.

[50] Benbow S, Humber F, Oliver TA, Oleson K, Raberinary D, Nadon M (2014) Lessons learnt from experimental temporary octopus fishing closures in south-west Madagascar: benefits of concurrent closures. Afr J Mar Sci 36: 31–37. 10.2989/1814232X.2014.893256

[51] Rodrigues Regional Assembly (2012) Octopus Closed Season Regulations (Session 2, 69). 69.

[52] Raberinary D, Benbow S (2012) The reproductive cycle of Octopus cyanea in southwest Madagascar and implications for fisheries management. Fish Res 125: 190–197. Available: http://www.sciencedirect.com/science/article/pii/S0165783612001051.

[53] van Heukelem W (1973) Growth and life-span of Octopus cyanea (Mollusca: Cephalopoda). J Zool 169: 299–315. Available: http://onlinelibrary.wiley.com/doi/10.1111/j.1469-7998.1973.tb04559.x/abstract.

[54] Roper CFE, Sweeney MJ, Nauen CE (1984) FAO Species Catalogue Vol 3. Cephalopods of the world. FAO Fish Synopses: 1–280.

[55] Guard M, Mgaya Y (2002) The artisanal fishery for Octopus cyanea Gray in Tanzania. Ambio 31: 528–536. Available: http://ambio.allenpress.com/perlserv/?request=get-abstract&doi=10.1639%2F0044-7447(2002)031%5B0528%3ATAFFOC%5D2.0.CO%3B2. 12572818

[56] Cooke A, Lutjeharms JRE, Vasseur P (2003) Marine and Coastal Ecosystems In: Goodman SM, Benstead JP, editors. The Natural History of Madagascar. Chicago: University of Chicago Press pp. 179–209.

[57] Barnes DK, Rawlinson KA (2009) Traditional coastal invertebrate fisheries in south-western Madagascar. J Mar Biol Assoc UK 89: 1589–1596. 10.1017/S0025315409000113

[58] Andriamalala G, Gardner C (2010) L’utilisation du dina comme outil de gouvernance des ressources naturelles: leçons tirés de Velondriake, sud-ouest de Madagascar. Tropical Conservation Science 3: 447–472.

[59] Astuti R (1995) People of the sea Cambridge University Press. 1 pp.

[60] INSTAT (2012) Pauvreté à Madagascar. Antananarivo, Madagascar: INSTAT 10.1111/cars.12058

[61] Barnes-Mauthe M, Oleson KLL, Zafindrasilivonona B (2013) The total economic value of small-scale fisheries with a characterization of post-landing trends: An application in Madagascar with global relevance. Fish Res 147: 175–185. 10.1016/j.fishres.2013.05.011

[62] Malleret-King D, Glass A, Wanyonyi I, Bunce L, Pomeroy B (2006) Socio-economic Monitoring guidelines for coastal managers of the Western Indian Ocean, SocMon WIO. CORDIO East Africa.

[63] Fink A (2003) The Survey Handbook. 2nd ed. Thousand Oaks, California: Sage Publications.

[64] Ratsimbazafy H (2010) Etude des effets biologiques des réserves marines temporaires sur la population des poulpes Octopus cyanea. Cas de APM Velondriake. Masters Thesis: Institut Halieutique et des Sciènces Marines.

[65] Bates D, Maechler M, Bolker B, Walker S, Eigen C (2013) Package “lme4.”

[66] R Core Team (2013) R: A Language and Environment for Statistical Computing. Available: http://www.R-project.org.

[67] Van Heukelem WF (1976) Growth, bioenergetics and life-span of Octopus cyanea and Octopus maya Honolulu, HI: University of Hawaii at Manoa.

[68] Holden ST, Shiferaw B, Wik M (1998) Poverty, market imperfections and time preferences: of relevance for environmental policy? Environ Dev Econ: 105–130.

[69] Harrison GW, Lau MI, Rutström EE, Sullivan MB (2005) Eliciting Risk And Time Preferences Using Field Experiments: Some Methodological Issues In: Isaac PRM, Norton DA, editors. Research in Experimental Economics. Research in Experimental Economics. Bingley: Emerald (MCB UP), Vol. 10 pp. 125–218. 10.1016/S0193-2306(04)10005-7

[70] Oleson KLL, Barnes-Mauthe M, Brander LM, Oliver TA, van Beek I, Zafindrasilivonona B, et al. (2015) Cultural bequest values for ecosystem service flows among indigenous fishers: A discrete choice experiment validated with mixed methods. Ecol Econ (In Press).

[71] Teh LS, Teh LC, Rashid Sumaila U (2014) Time preference of small-scale fishers in open access and traditionally managed reef fisheries. Marine Policy 44: 222–231.

[72] Tucker B (2012) Do risk and time experimental choices represent individual strategies for coping with poverty or conformity to social norms? Evidence from rural southwestern Madagascar. Curr Anthropol: 1–38. Available: message:%3C2CCA5486-FF3F-46AE-8A62-3FDCBC5C7ACD@uga.edu%3E.

[73] Chabris CF, Laibson D, Morris CL, Schuldt JP, Taubinsky D (2008) Individual laboratory-measured discount rates predict field behavior. J Risk Uncertain 37: 237–269. 10.1007/s11166-008-9053-x 19412359 PMC2676104

[74] Costanza R, Daly HE (1992) Natural capital and sustainable development. Conserv Biol 6: 37–46.

[75] Hardisty DJ, Weber EU (2009) Discounting future green: Money versus the environment. J Exp Psychol Gen 138: 329 10.1037/a0016433 19653793

[76] Otero J, Rocha F, González ÁF, Gracia J, Guerra Á (2005) Modelling artisanal coastal fisheries of Galicia (NW Spain) based on data obtained from fishers: the case of Octopus vulgaris. Sci Mar 69: 577–585.

[77] Quetglas A, Alemany F, Carbonell A, Merella P, Sánchez P (1998) Biology and fishery of Octopus vulgaris Cuvier, 1797, caught by trawlers in Mallorca (Balearic Sea, Western Mediterranean). Fish Res 36: 237–249.

[78] Herwig JN, Depczynski M, Roberts JD, Semmens JM, Gagliano M, Heyward AJ (2012) Using age-based life history data to investigate the life cycle and vulnerability of Octopus cyanea. PLoS ONE 7: e43679 10.1371/journal.pone.0043679 22912898 PMC3422261

[79] Gough C, Thomas T, Humber F, Harris A, Cripps G, Peabody S (2009) Vezo fishing: an introduction to the methods used by fishers in Andavadoaka southwest Madagascar. Blue Ventures Conservation Report Blue Ventures, UK Available at.

[80] Blumberg R (1988) Income under female versus male control. J Fam Issues 9: 51–84. Available: http://jfi.sagepub.com/content/9/1/51.short. 12281311 10.1177/019251388009001004

[81] Ribot J C, Peluso N L (2003), A Theory of Access. Rural Sociology, 68: 153–181. 10.1111/j.1549-0831.2003.tb00133.x

[82] Barnes-Mauthe M, Oleson KLL, Brander LM, Zafindrasilivonona B, Oliver TA, van Beukering P (2015) Social capital as an ecosystem service: Evidence from a locally managed marine area. Ecosystem Services. (in press) 10.1016/j.ecoser.2014.10.009

[83] Ostrom E, Janssen MA, Anderies JM (2007) Going beyond panaceas. Proc Natl Acad Sci U S A 104: 15176–15178. 10.1073/pnas.0701886104 17881583 PMC2000490

