# Supplementary figures and images for: Positive Catch & Economic Benefits of Periodic Octopus Fishery Closures: Do Effective, Narrowly Targeted Actions ‘Catalyze’ Broader Management?
Source: PLoS One. 2015 Jun 17;10(6):e0129075. doi: 10.1371/journal.pone.0129075 (PMC4471298; doi:10.1371/journal.pone.0129075)

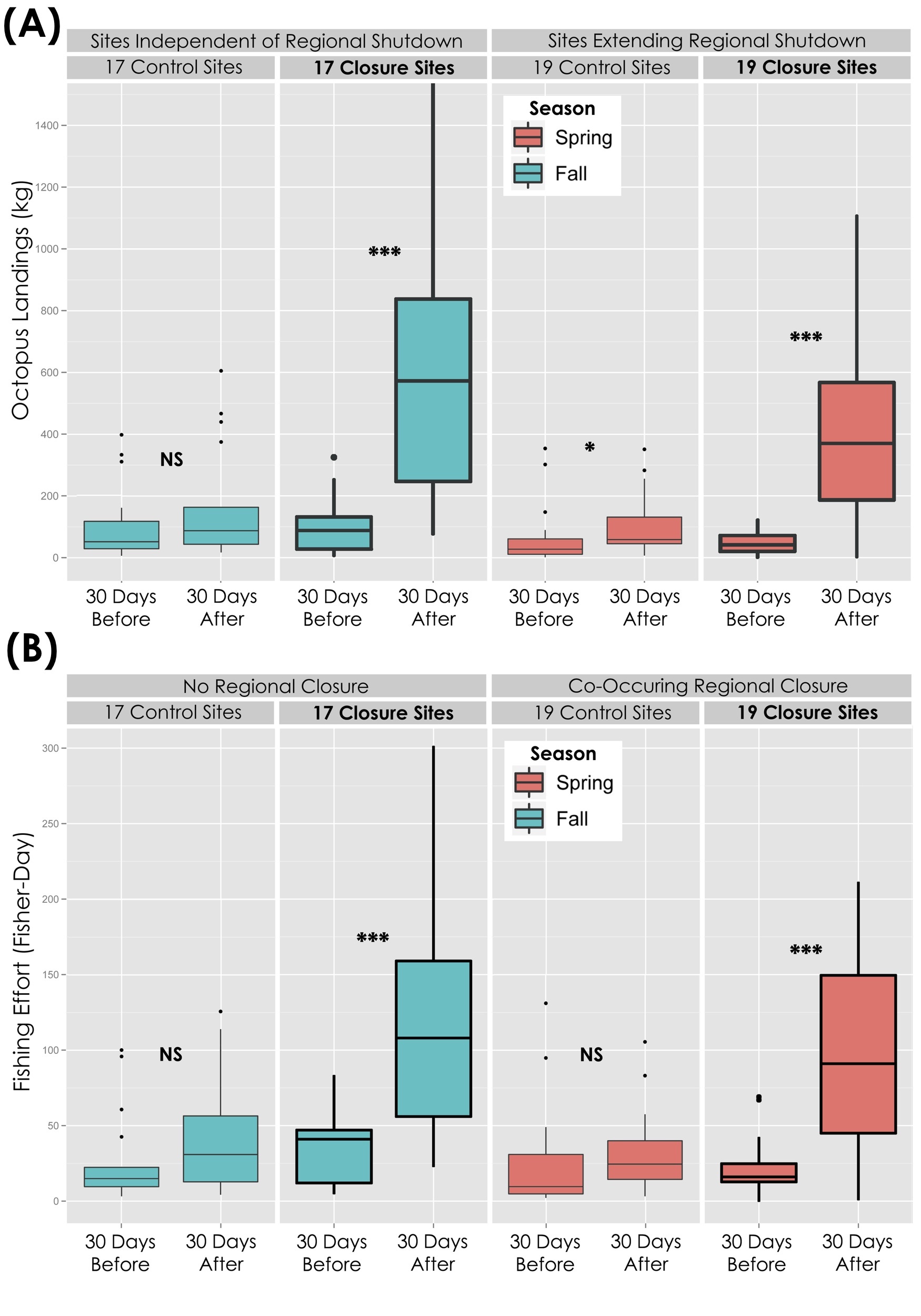

Supplement: S1 Fig — Site-level (a) octopus landings (kg) and (b) fishing effort (fisher-days), 30 days before and after closure at closed sites and paired control sites. Data are separated by season, thus separating those closures that occurred independently of a regional fishery shutdown (“no ban”, 17/36), and those that extended the shutdown (“ban”, 19/36). Significance indicators show distinctions between a particular group and its “before” group comparison, independent contrasts from linear mixed models. NS = Not Significant; * = p<0.05; *** = p < 0.001. Seasons indicate timing of closure reopening, Spring = February-May, Fall = June-September. (JPG) [file pone.0129075.s001.jpg]

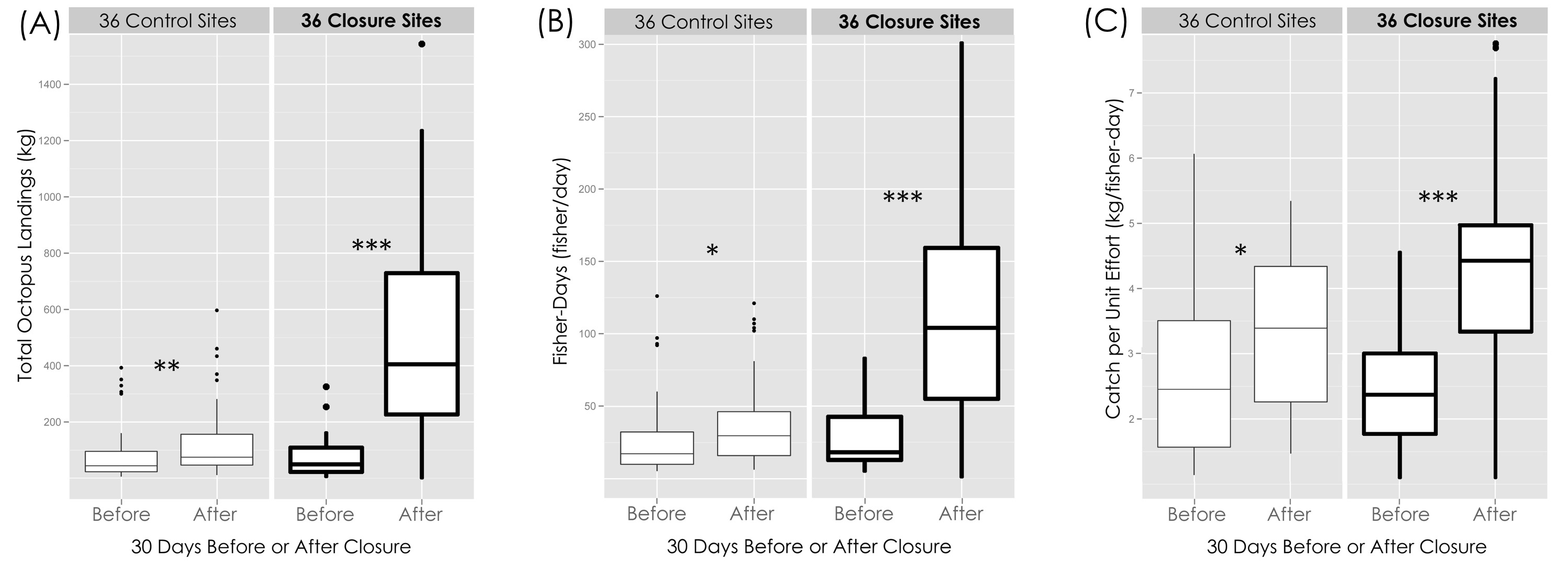

Supplement: S2 Fig — Site-level (a) octopus landings (kg), (b) fishing effort (fisher-days), and (c) catch-per-unit effort (kg/fisher-day), 30 days before and after closure at closed sites and paired control sites. Data are aggregated across season/fishery shutdown. Significance indicators show distinctions between a particular group and its “before” group comparison, independent contrasts from linear mixed models. NS = Not Significant; * = p<0.05; ** = p<0.01; *** = p < 0.001. (JPG) [file pone.0129075.s002.jpg]

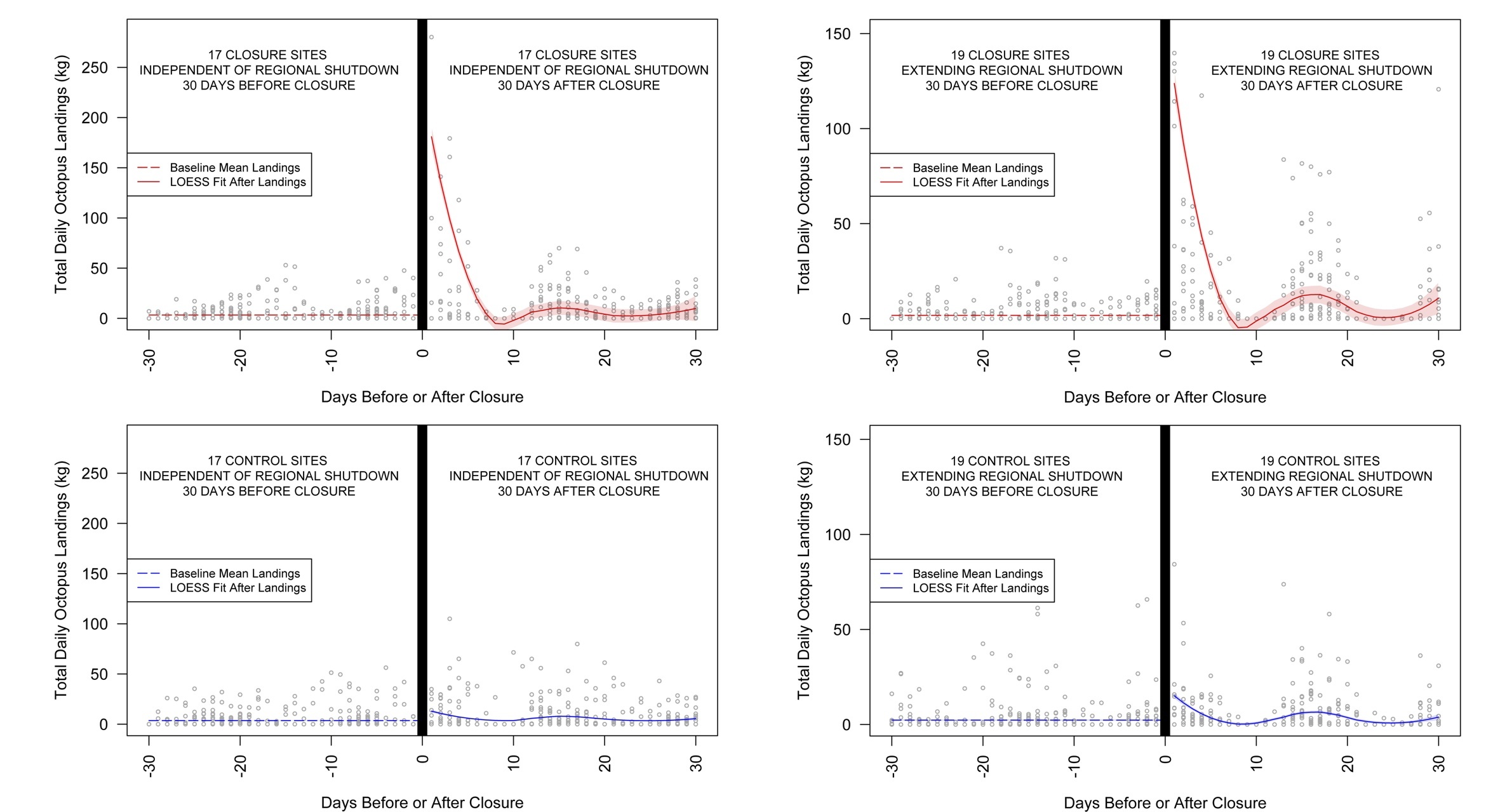

Supplement: S3 Fig — Site-level octopus landings (kg) 30 days before and after closure at closed sites (upper, red) and paired control sites (lower, blue). Data are separated by season, thus separating those closures that occurred independently of a regional fishery shutdown (“no ban”, 17/36), and those that extended the shutdown (“ban”, 19/36). Raw daily totals are shown as gray points, with an overall mean and 95% CI shown as a red/blue line ‘before’, and a LOESS fit with 95%CI shown ‘after’. Note that due to timing the opening with the low spring tide, data ‘after’ are in phase with tidal cycles, while those ‘before’ are not. (JPG) [file pone.0129075.s003.jpg]

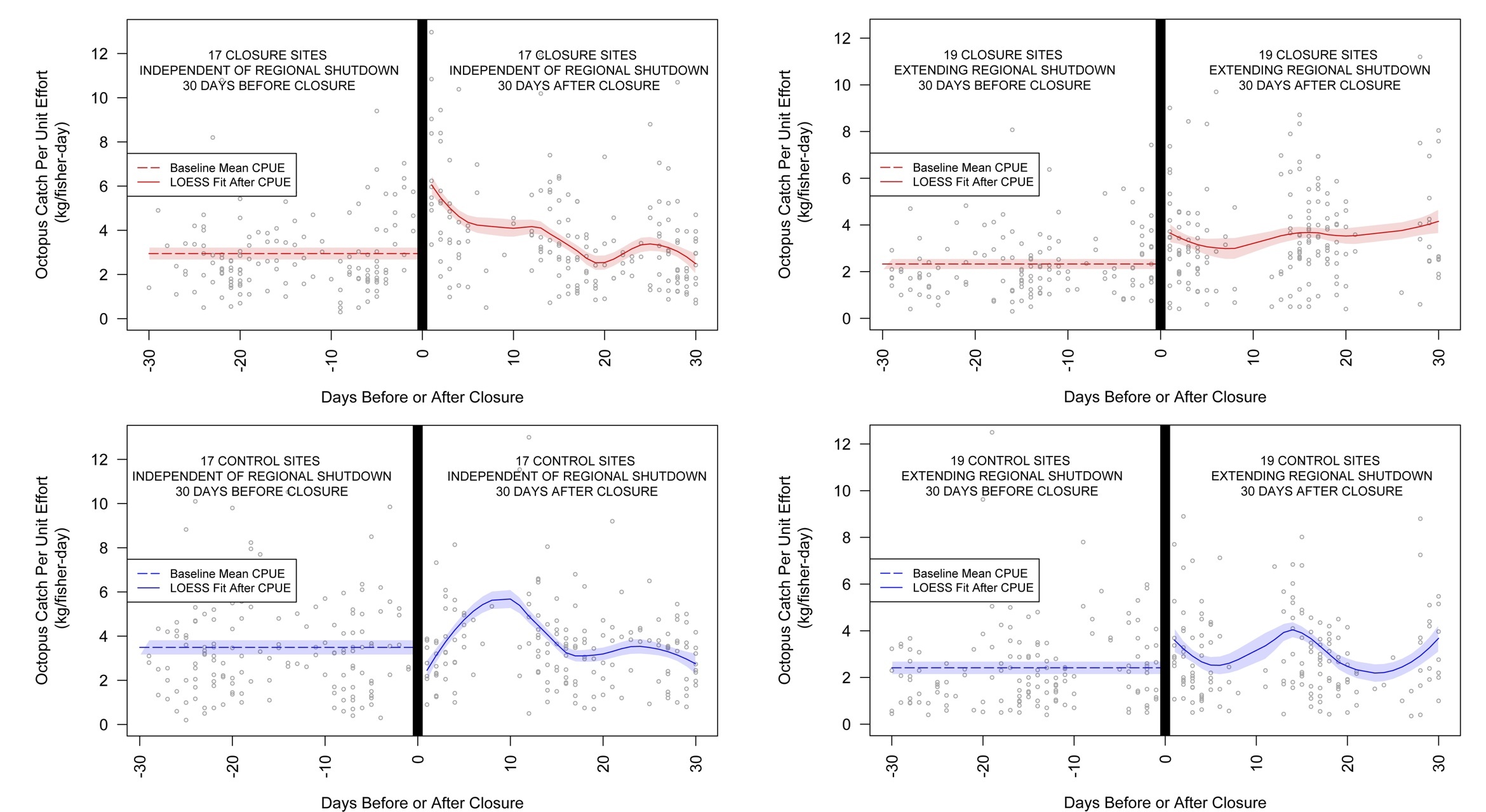

Supplement: S4 Fig — Site-level catch-per-unit-effort (kg/fisher-day) 30 days before and after closure at closed sites (upper, red) and paired control sites (lower, blue). Data are separated by season, thus separating those closures that occurred independently of a regional fishery shutdown (“no ban”, 17/36), and those that extended the shutdown (“ban”, 19/36). Raw daily totals are shown as gray points, with an overall mean and 95% CI shown as a red/blue line ‘before’, and a LOESS fit with 95%CI shown ‘after’. Note that due to timing the opening with the low spring tide, data ‘after’ are in phase with tidal cycles, while those ‘before’ are not. (JPG) [file pone.0129075.s004.jpg]

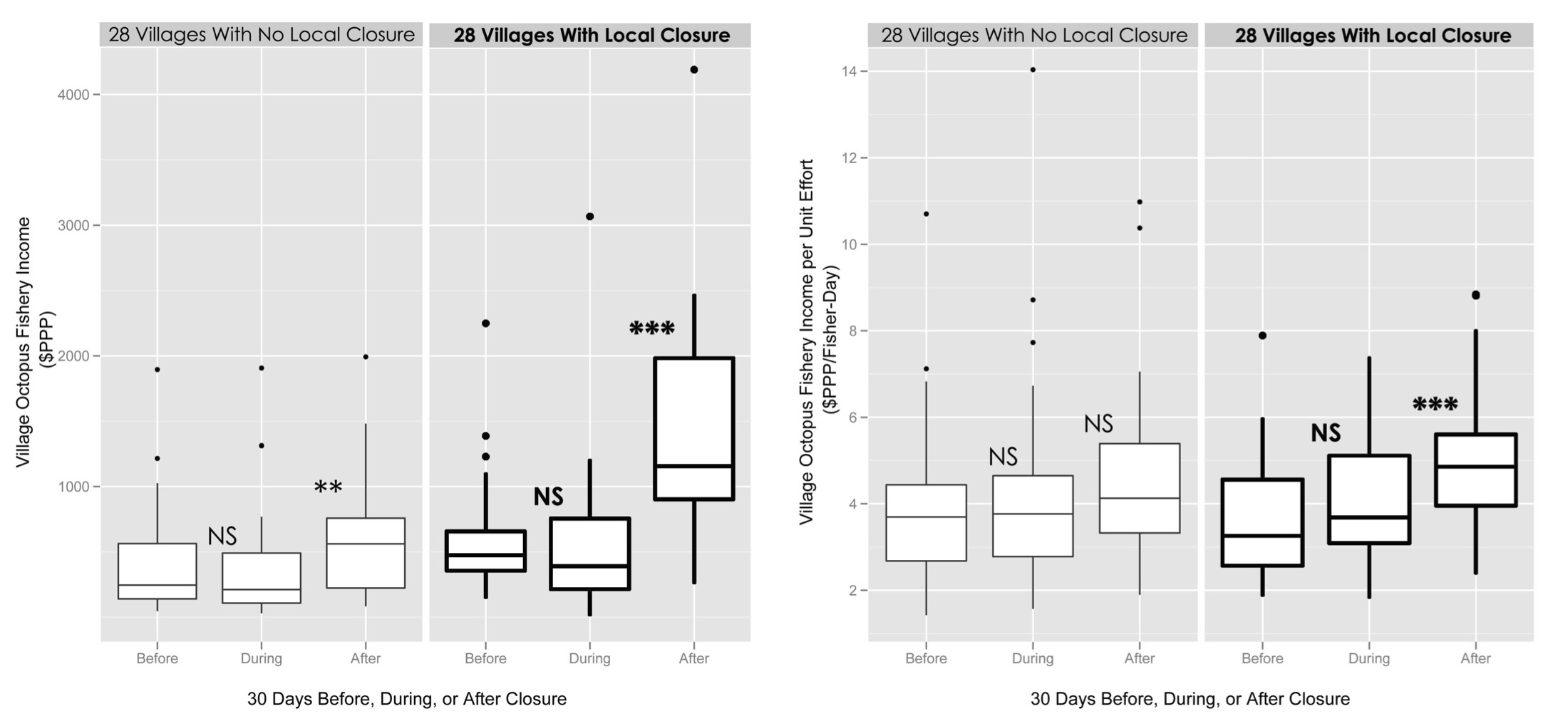

Supplement: S5 Fig — Total village-level octopus fishing income ($PPP), and village-level octopus fishing income per unit of fishing effort ($PPP/fisher-day) 30 days before, during, and after closures, at villages both with and without closures. The data depicted are from 28 closure periods showing closure-sponsoring villages and their paired control villages from 2004–2011. Data are aggregated by season. As “during” periods are not exactly 30 days, “during” values are scaled to a per-30-day measure. Significance indicators show distinctions between a particular group and its “before” group comparison, from linear mixed-effect model. NS = Not Significant; * = p < 0.05; ** = p < 0.01; *** = p < 0.001. (JPG) [file pone.0129075.s005.jpg]

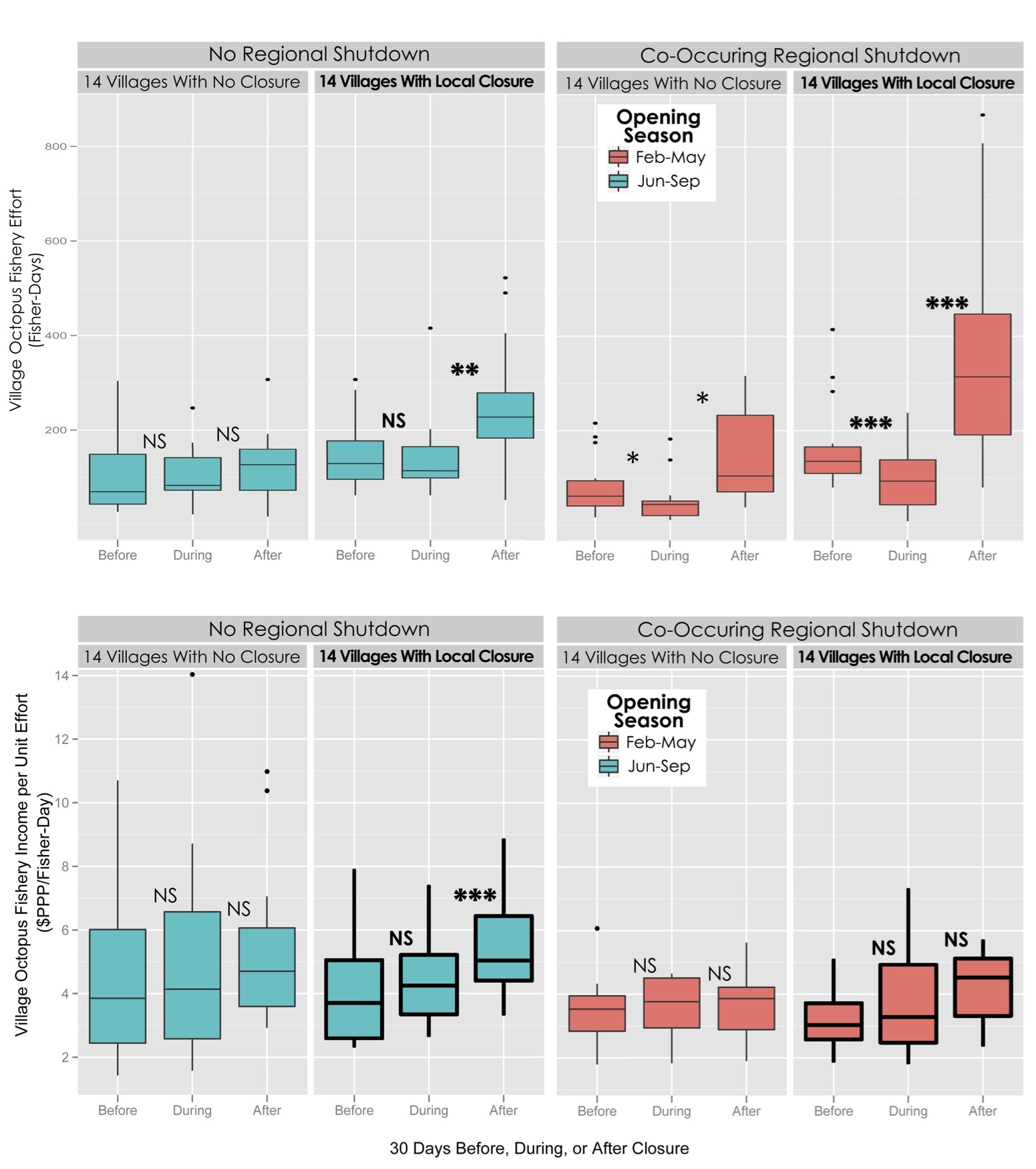

Supplement: S6 Fig — Total village-level octopus fishing effort (fisher-days) and village-level octopus fishing income per unit effort ($PPP/fisher-day) 30 days before, during, and after closures, at villages both with and without closures. The data depicted are from 28 closure periods showing closure-sponsoring villages and their paired control villages from 2004–2011. Data are separated by season, thus separating those closures that extended a regional fishery shutdown, and those that occurred independently of the shutdown. As “during” periods are not exactly 30 days, “during” values are scaled to a per-30-day measure. Significance indicators show distinctions between a particular group and its “before” group comparison, from linear mixed-effect model. NS = Not Significant; * = p < 0.05; ** = p < 0.01; *** = p < 0.001. (JPG) [file pone.0129075.s006.jpg]

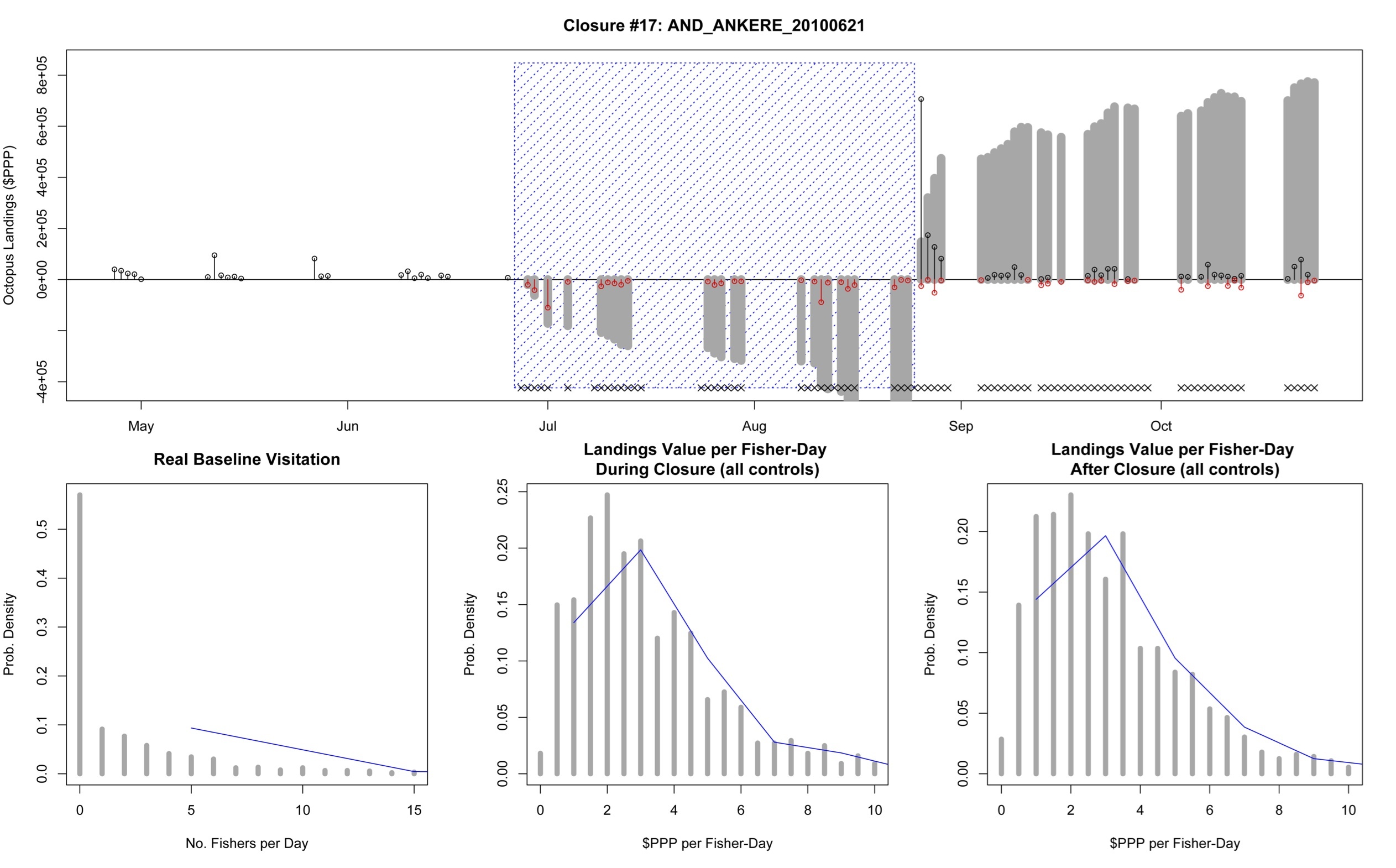

Supplement: S7 Fig — Here we plot an example of a single site in our site-based cost model, showing a timeline of the closure, with closure period (blue hatching), presence of fishing in nearby villages (x’s along bottom), actual recorded landings (i.e. closure ‘benefits’, black circles/bars), modeled foregone landings (i.e. closure ‘costs’, red circles/bars), and running sums (gray bars). Below we see the three distributions from which the counter-factual landings are simulated: the pattern of visitation at this site across the whole dataset (excluding data from closure and post-closure periods), and value-per-unit effort distributions both during and 60-days after the closure (again, excluding data from closure and post-closure periods). (JPG) [file pone.0129075.s007.jpg]

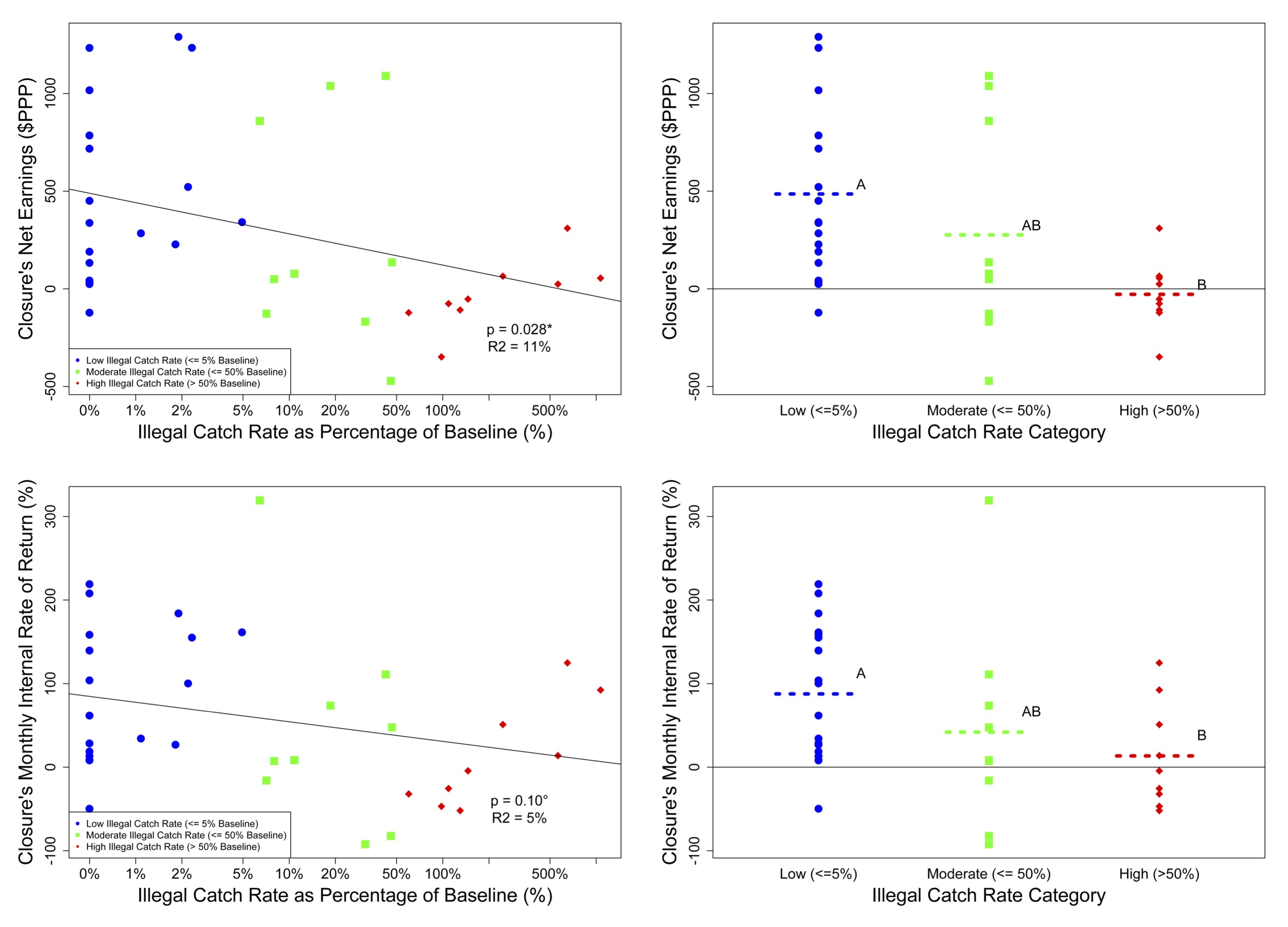

Supplement: S8 Fig — Here we compare a site’s modeled Net Earnings and monthly IRR to Illegal Catch Rates expressed as reported landings from a closed site during the closure as a percentage of baseline landings during the 60 days before closure. Comparisons among both NE & IRR, and Illegal catch rate are shown both as continuous regressions, and across catch rate categories. (JPG) [file pone.0129075.s008.jpg]

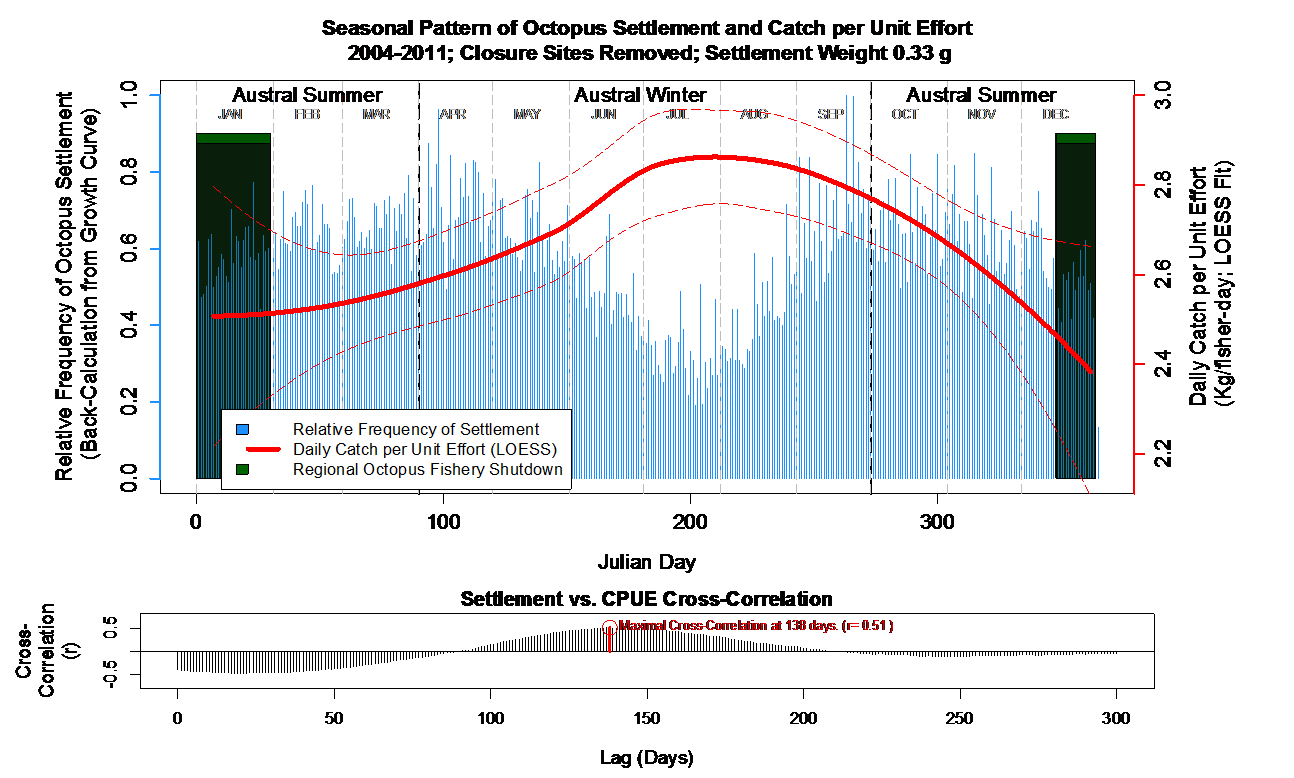

Supplement: S9 Fig — Here we plot frequency of octopus settlement (as back-calculated from individual mass at capture and a species-level growth curve) with Julian day, including data from 2004–2011 (blue bars). Overlaid on these data is a LOESS fit of seasonal CPUE variation with 95%CI (red lines), with closure sites removed. In green we show the timing of the annual regional fishery shutdown, which began in 2006. Note the increase in CPUE variability during this more data-poor time. Below we plot the cross-correlation of these trends, showing a maximal correlation at 159 days. (PNG) [file pone.0129075.s009.png]
