# Supplementary material for: Positive Catch & Economic Benefits of Periodic Octopus Fishery Closures: Do Effective, Narrowly Targeted Actions ‘Catalyze’ Broader Management?
Source: PLoS One. 2015 Jun 17;10(6):e0129075. doi: 10.1371/journal.pone.0129075 (PMC4471298; doi:10.1371/journal.pone.0129075)
Supplement: S1 File — (DOCX) [file pone.0129075.s010.docx]

# Supplementary Results

# Socioeconomic results

Overall, 25.3% of households in our target population (i.e., excluding inland villages) were sampled (Table S1). Sampling was fairly balanced across habitats and geographic regions (24% of total households in coastal habitat, 31% in island, 24% in mangrove; 32% of households in northern region, 26% in central region, and 19% in southern region). The total number of households in 2010 was based on censuses and surveys undertaken in 2006 across Velondriake’s 24 villages, which estimated a population size of 5,925 in 1,243 households [1,2]. Assuming a population growth of 2.95% [3] and using information from key informant interviews about household relocations, the total population in 2010 was 7,563 in 1,354 households; 1,186 excluding inland villages (applying a stratum-specific average household size that ranged from 4.64 to 7.71 with a mean of 5.58, as determined by this survey) (Table S2).

Households are poor, and as expected there is variation between regions and habitats (Table S3). Figures in the table are expressed in Malagasy Ariary (MGA) the official currency; according to the World Bank, 1 USD = 2,025 MGA in 2011, and $1 PPP = 1,142 MGA.

Per capita income is thus well below the $2 per day PPP poverty line in every quadrant except for island villages (Table S4), and across all quadrants, 81% of households lived below $2 per person per day. Households were highly dependent on fishing and gleaning for their income (average 82% of household income), and on other natural resource-dependent activities, such as tourism, lime or charcoal production, mariculture, or the curio trade (an additional 4% of household income).

Few households ate octopus, on average, only 2% of a household’s weekly meals featured octopus.

**References:**

1. Epps M (2008) A Socioeconomic Baseline Assessment: Implementing the socioeconomic monitoring guidelines in southwest Madagascar. Blue Ventures Conservation Report.

2. Langley J, Harris A, Nihalani N (2006) The 2004-2005 census of Andavadoaka, southwest Madagascar. Blue Ventures Conservation Report. 29 pp.

3. INSTAT (2012) Pauvreté à Madagascar. Antananarivo, Madagascar: INSTAT.
