# Supplementary material for: Positive Catch & Economic Benefits of Periodic Octopus Fishery Closures: Do Effective, Narrowly Targeted Actions ‘Catalyze’ Broader Management?
Source: PLoS One. 2015 Jun 17;10(6):e0129075. doi: 10.1371/journal.pone.0129075 (PMC4471298; doi:10.1371/journal.pone.0129075)
Supplement: S1 Table — (DOCX) [file pone.0129075.s011.docx]

**Table S1. Estimated population and number of households and household survey sample size by stratum**

| Region | Estimated Population | Estimated # of Households | Household Survey Sample | % Sampled |
| --- | --- | --- | --- | --- |
| North Island | 758 | 156 | 37 | 23.7% |
| North Coastal | 631 | 135 | 40 | 29.6% |
| North Mangrove | 489 | 74 | 40 | 54.1% |
| Central Island | 433 | 83 | 37 | 44.6% |
| Central Coastal | 1926 | 280 | 42 | 15.0% |
| Central Mangrove | 151 | 24 | 23 | 95.8% |
| South Coastal ^*^ | 471 | 94 | 40 | 42.6% |
| South Mangrove | 1,800 | 342 | 42 | 12.3% |
| **Total** | **6,659** | **1,188** | **301** | 25.3% |
| Inland ^†^ | 904 | 168 | - |  |

^*^ No South-Island villages exist.

^†^ Excluded from survey but within Velondriake’s boundaries.
