# Supplementary material for: Positive Catch & Economic Benefits of Periodic Octopus Fishery Closures: Do Effective, Narrowly Targeted Actions ‘Catalyze’ Broader Management?
Source: PLoS One. 2015 Jun 17;10(6):e0129075. doi: 10.1371/journal.pone.0129075 (PMC4471298; doi:10.1371/journal.pone.0129075)
Supplement: S2 Table — (DOCX) [file pone.0129075.s012.docx]

**Table S2. Village characteristics: stratum, population, and households**

| **Village name** | **Habitat** | **Region** | **Population 2006** | **# HH 2006** | **Population 2010** | **# HH in 2010** |
| --- | --- | --- | --- | --- | --- | --- |
| *Ambalorao* | *Inland* | *South* | *105* | *14* | *118* | *22* |
| *Ankilimalinike* | *Inland* | *South* | *84* | *15* | *95* | *18* |
| *Befandefa* | *Inland* | *South* | *614* | *146* | *691* | *128* |
| Ankitambagna | Coastal | South | 86 | 16 | 97 | 19 |
| Tampolove | Coastal | South | 311 | 61 | 350 | 70 |
| Tsimivolo | Coastal | South | 21 | 3 | 24 | 4 |
| Agnolignoly | Mangrove | South | 234 | 50 | 263 | 49 |
| Ampasimara | Mangrove | South | 125 | 25 | 141 | 26 |
| Ankindranoke | Mangrove | South | 448 | 85 | 504 | 93 |
| Lamboara | Mangrove | South | 526 | 106 | 612 | 122 |
| Vatoavo | Mangrove | South | 249 | 52 | 280 | 50 |
| Ampasilava | Coastal | Central | 321 | 63 | 509 | 96 |
| Andavadoaka | Coastal | Central | 1,220 | 233 | 1,419 | 184 |
| Andambatihy | Island | Central | 72 | 16 | 173 | 37 |
| Nosy Hao | Island | Central | 259 | 62 | 86 | 16 |
| Nosy Mitata | Island | Central | 131 | 25 | 39 | 7 |
| Nosy Ve | Island | Central | 119 | 22 | 134 | 23 |
| Antsatsamoroy | Mangrove | Central | 90 | 14 | 101 | 16 |
| Antseranangy | Mangrove | Central | 44 | 7 | 50 | 9 |
| Ambolimoke | Coastal | Northern | 89 | 20 | 100 | 18 |
| Bevato | Coastal | Northern | 472 | 93 | 531 | 117 |
| Andragnombala | Island | Northern | 150 | 27 | 169 | 35 |
| Nosy Be | Island | Northern | 523 | ND | 589 | 121 |
| Belavenoke | Mangrove | Northern | 435 | 88 | 489 | 74 |
| **Sampled Velondriake**  **(excluding inland)** | | | **5,925** | **1,068** | **6,659** | **1,186** |
| **All Velondriake (including inland)** | | | **6,728** | **1,243** | **7,563** | **1,354** |
