# Supplementary material for: Positive Catch & Economic Benefits of Periodic Octopus Fishery Closures: Do Effective, Narrowly Targeted Actions ‘Catalyze’ Broader Management?
Source: PLoS One. 2015 Jun 17;10(6):e0129075. doi: 10.1371/journal.pone.0129075 (PMC4471298; doi:10.1371/journal.pone.0129075)
Supplement: S3 Table — (DOCX) [file pone.0129075.s013.docx]

**Table S3. Average household incomes across regions and habitats (2011 MGA per week)**

|  | Coast | Island | Mangrove | **Average** |
| --- | --- | --- | --- | --- |
| North | 44,869 | 90,597 | 59,541 | 67,387 |
| Central | 82,467 | 70,680 | 58,290 | 78,440 |
| South | 53,546 | NA | 40,358 | 43,201 |
| **Average ^a^** | 73,094 | 83,855 | 45,076 | **62,111** |

^a^ Average is based on a number of household-weighted average.
