# Supplementary material for: Positive Catch & Economic Benefits of Periodic Octopus Fishery Closures: Do Effective, Narrowly Targeted Actions ‘Catalyze’ Broader Management?
Source: PLoS One. 2015 Jun 17;10(6):e0129075. doi: 10.1371/journal.pone.0129075 (PMC4471298; doi:10.1371/journal.pone.0129075)
Supplement: S4 Table — (DOCX) [file pone.0129075.s014.docx]

**Table S4. Average daily per capita income across regions and habitats (2011 PPP per person per day)**

|  | Coast | Island | Mangrove | Average |
| --- | --- | --- | --- | --- |
| North | 2.05 | 3.16 | 1.42 | 2.34 |
| Central | 1.71 | 2.06 | 1.45 | 1.75 |
| South | 1.51 | NA | 1.09 | 1.18 |
| Average^b^ | 1.74 | 2.76 | 1.18 | **1.72** |

^b^Average is based on a population-weighted average.
