# Supplementary material for: Positive Catch & Economic Benefits of Periodic Octopus Fishery Closures: Do Effective, Narrowly Targeted Actions ‘Catalyze’ Broader Management?
Source: PLoS One. 2015 Jun 17;10(6):e0129075. doi: 10.1371/journal.pone.0129075 (PMC4471298; doi:10.1371/journal.pone.0129075)
Supplement: S5 Table — (DOCX) [file pone.0129075.s015.docx]

**Table S5. Fishers’ octopus sale prices**

| **Year** | **Northern** | **Central** | **Southern** | **Average** | **CPI** | **Average** | **Average** |
| --- | --- | --- | --- | --- | --- | --- | --- |
|  | (MGA / kg) | (MGA / kg) | (MGA / kg) | (nominal MGA / kg) | (%) | (2011 MGA / kg) | ($ 2011 PPP / kg) |
| 2003 |  | 400 |  | 400 | 2.8 | 867 | 0.76 |
| 2004 |  | 800 |  | 800 | 14.3 | 1,687 | 1.48 |
| 2005 |  | 1,000^*^ |  | 1,000 | 18.3 | 1,845 | 1.62 |
| 2006 | 2,000 | 1,300 |  | 1,650 | 11.5 | 2,573 | 2.25 |
| 2007 | 1,600 | 1,300 | 1,200 | 1,367 | 9.6 | 1,911 | 1.67 |
| 2008 | 1,000 | 1,000 | 1,200^†^ | 1,067 | 8.9 | 1,361 | 1.19 |
| 2009 | 1,000 | 1,000 | 1,000 | 1,000 | 8.4^§^ | 1,172 | 1.03 |
| 2010 | 1,000 | 1,000 | 1,000 | 1,000^‡^ | 8.1^§^ | 1,081 | 0.95 |
| 2011 | 1,000 | 1,000 | 1,000 | 1,000^‡^ |  | 1,000 | 0.88 |
|  |  |  |  |  | **Mean** | **1,500** | **1.31** |

^*^ Price rose to 1,200 MGA at some point during year

^†^ Price rose to 1,500 then dropped to 1,000 during the year

^‡^ In 2009 and 2010, octopuses over 2 kg caught on the opening day of the closure were worth 1,400 MGA a kilogram

§ Since our initial analysis (2011), these numbers have been retro-actively updated to 9.2 and 9.3, respectively. Our analysis reflects the 2011 numbers shown here.
